# Activation of DNMT1 by Chemically Synthesized Dual‐Monoubiquitinated PAF15 Protein

**DOI:** 10.1002/advs.78636

**Published:** 2026-11-10

**Authors:** Sae Suzuki, Yuya Takahashi, Amika Kikuchi, Satomi Kori, Fumika Yoshitomi, Hiroshi Murakami, Kyohei Arita, Gosuke Hayashi

**Affiliations:** ^1^ Department of Biomolecular Engineering Graduate School of Engineering Nagoya University Nagoya Japan; ^2^ Graduate School of Medical Life Science Yokohama City University Yokohama Kanagawa Japan; ^3^ Institute of Nano‐Life Systems Institutes of Innovation for Future Society Nagoya University Nagoya Japan; ^4^ Research Institute for Quantum and Chemical Innovation Institutes of Innovation for Future Society Nagoya University Nagoya Japan; ^5^ Institute of Advanced Medical Sciences Tokushima University Tokushima Japan; ^6^ Institute for Life and Medical Sciences Kyoto University Kyoto Japan; ^7^ Department of Chemical Science and Engineering Graduate School of Engineering Kyoto University Kyoto Japan

**Keywords:** chemical protein synthesis, Cryo‐EM, DNA methylation maintenance, ubiquitinated protein

## Abstract

Replication‐coupled DNA methylation maintenance by DNA methyltransferase 1 (DNMT1) is mediated by dual‐monoubiquitinated PCNA‐associated factor 15 (PAF15Ub2). However, the molecular mechanism of DNMT1 activation by PAF15Ub2 remains unclear. Here, taking advantage of chemical protein synthesis, we prepare PAF15 protein bearing dual‐monoubiquitination through native amide linkages and determine the active conformation of DNMT1 complexed with the synthetic PAF15Ub2 and hemimethylated DNA by cryogenic electron microscopy (cryo‐EM). With an optimized convergent synthetic route, the 262 amino‐acid protein PAF15Ub2 was assembled from six synthetic peptide segments, including two branched peptides. By employing a one‐pot ligation technique and deselenization chemistry, we obtained the native sequence of PAF15Ub2 in high purity and good yield. The chemically synthesized PAF15Ub2 was shown to bind to DNMT1 and stimulate its methyltransferase activity in biochemical assays. Cryo‐EM analysis of DNMT1 in complex with PAF15Ub2 and hemimethylated DNA revealed that DNMT1 adopts an active conformation in which the RFTS domain is released from the catalytic domain, thereby relieving the autoinhibition.

## Graphical abstract

DNA methyltransferase 1 (DNMT1) propagates DNA methylation patterns during replication and is stimulated by dual‐monoubiquitinated PCNA‐associated factor 15 (PAF15Ub2). We chemically synthesized PAF15Ub2 with native isopeptide linkages. Cryo‐EM analysis of DNMT1 bound to the synthetic PAF15Ub2 and hemimethylated DNA revealed an active conformation with the autoinhibitory replication foci targeting sequence (RFTS) domain released from the catalytic domain.

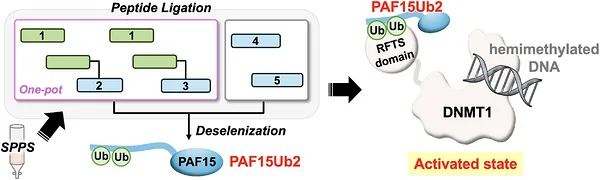

## 1 Introduction

Cytosine methylation at CpG dinucleotides is involved in diverse biological processes in mammals, such as retrotransposon silencing, X‐chromosome inactivation, genomic imprinting and carcinogenesis [1, 2]. Maintenance DNA methylation is a fundamental mechanism that ensures the faithful inheritance of epigenetic information to daughter cells. After DNA replication, the hemimethylated DNA duplex, which contains methylated and unmethylated cytosines in the parental and nascent strands, respectively, is recognized by the DNA methylation machinery and eventually converted to the fully methylated state. The enzyme responsible for this maintenance methylation is DNA methyltransferase 1 (DNMT1) [3], which contains multiple domains including a replication foci targeting sequence (RFTS), an unmethylated CpG DNA‐binding CXXC domain, bromo‐adjacent homology (BAH) domains and a C‐terminal catalytic domain [4]. Apo‐form DNMT1 adopts an autoinhibitory conformation, in which the RFTS domain occludes the DNA binding surface of the catalytic domain, thereby preventing DNMT1 recruitment to hemimethylated DNA and aberrant DNA methylation [5, 6, 7]. The DNA methyltransferase activity is restored upon binding of the RFTS domain to dual‐monoubiquitinated histone H3 at K18 and K23 (H3Ub2) [8, 9], which is accompanied by conformational changes in DNMT1 [10, 11]. DNMT1 binding to H3Ub2 has also been demonstrated at the nucleosome level using H3Ub2 mimics [12]. The dual‐monoubiquitination of H3 is essential for maintenance DNA methylation and is catalyzed by the E3 ubiquitin ligase ubiquitin‐like with PHD and RING finger domains 1 (UHRF1) [8, 9, 13, 14], which recognizes hemimethylated DNA via its SET and RING‐associated (SRA) domain [15, 16, 17]. UHRF1 also catalyzes the dual ubiquitination of PCNA‐associated factor 15 (PAF15) at K15 and K24 [18, 19] during early DNA replication. DNMT1 is recruited to DNA methylation sites through interaction of its RFTS domain with the dual‐monoubiquitinated PAF15 (PAF15Ub2), thereby promoting the maintenance of DNA methylation (Figure 1) [20, 21]. A recently reported study attempted to reconstitute the DNMT1:PAF15Ub2:PCNA:hemimethylated DNA complex for structural analysis [22]. The formation of the quaternary complex was demonstrated by mass photometry and negative‐stain electron microscopy, but the complex dissociated upon vitrification, and the negative‐stain reconstruction was too low in resolution (24 Å) to place the individual components. An AlphaFold model was therefore proposed, which suggested that PAF15Ub2 may convert DNMT1 into its active conformation as observed for H3Ub2. The structural basis of DNMT1 activation by PAF15Ub2 has thus remained experimentally unresolved. To obtain mechanistic insight into this activation, we sought to perform biochemical and structural analyses of DNMT1 activation by PAF15Ub2. In our preliminary cryogenic electron microscopy (cryo‐EM) analysis, we found that DNMT1 aggregates under non‐reducing conditions. However, the previously developed PAF15Ub2 mimic relies on disulfide linkages [20], which easily degrade under reducing conditions. We therefore sought to prepare PAF15 bearing two ubiquitin moieties connected via native isopeptide bonds. Although several ubiquitinated protein mimics that are stable under reducing conditions have been reported [23, 24], the most faithful way to recapitulate protein function is to use proteins with native linkages.

**FIGURE 1:**
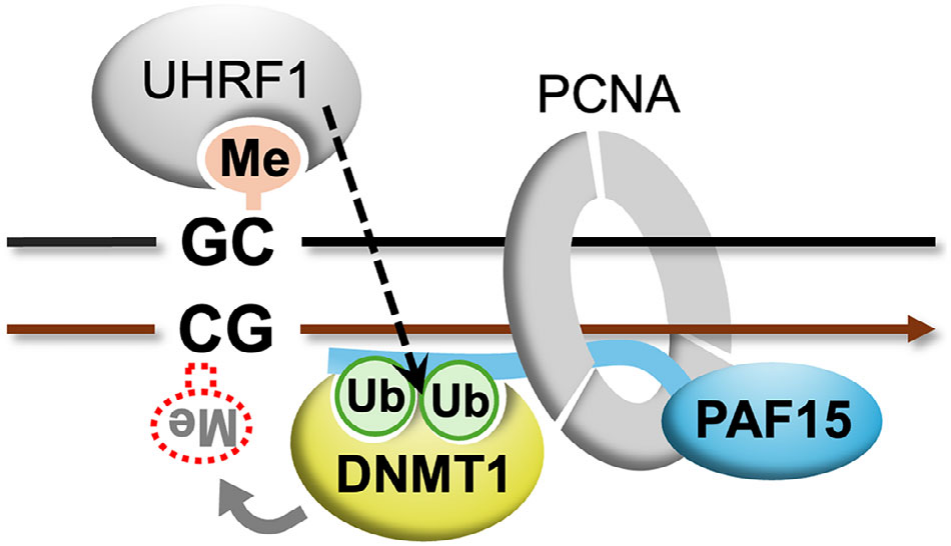
Schematic model of the PAF15Ub2‐mediated maintenance DNA methylation.

Ubiquitinated proteins can be prepared enzymatically using a ubiquitin‐activating enzyme (E1), a ubiquitin‐conjugating enzyme (E2) and a ubiquitin ligase (E3). Among these, the E2 and E3 enzymes determine the substrate specificity of ubiquitination and therefore must be identified for each target protein. For example, the recombinant E2 enzyme UBE2T and E3 ligase FANCL have been shown to monoubiquitinate a specific substrate, the FANCI–FANCD2 (ID2) complex, in vitro [25, 26] and the resulting ubiquitinated products have served as valuable tools in subsequent studies [27, 28, 29]. In another case, the recombinant E2 enzyme UbcH5 and E3 ligase RNF8 have been shown to stoichiometrically monoubiquitinate the homotrimeric PCNA complex in vitro [30], providing homogeneous ubiquitinated PCNA that enabled its X‐ray crystallographic and biochemical characterization [31]. However, the in vitro enzymatic preparation of ubiquitinated proteins may be problematic in some cases, in part because it requires the prior identification and isolation of target‐specific E2 and E3 enzymes. The enzymatic reactions often proceed with low site selectivity, modifying unintended lysine residues in addition to the target lysine and thereby generating multi‐monoubiquitinated species. Furthermore, polyubiquitinated products are frequently formed and unreacted substrate often remains, making it difficult to isolate homogeneous monoubiquitinated proteins [32].

Chemical protein synthesis, in which peptide segments prepared by solid‐phase peptide synthesis (SPPS) or recombinant expression are chemically assembled, provides an alternative approach to access site‐specifically ubiquitinated proteins with native structures. The assembly is most commonly achieved by native chemical ligation (NCL) [33], in which a new peptide bond forms between a C‐terminal peptide thioester and an N‐terminal cysteinyl or selenocysteinyl peptide. Because these residues can subsequently be converted to alanine by desulfurization [34] or deselenization [35], the native sequence is restored at the ligation junctions. In the case of ubiquitinated proteins, the preparation of ubiquitinated peptide segments is not straightforward, since single‐shot SPPS is difficult due to their large size (e.g.,> 80–100 aa). Sophisticated methodologies such as auxiliary‐mediated chemical ubiquitination [36, 37] and the use of preformed isopeptide‐linked ubiquitin building blocks [38] have therefore been developed so far. Furthermore, the synthesis of large proteins (e.g.,> 200 aa), such as ubiquitinated proteins, requires a greater number of peptide segments, leading to an increased number of ligation steps and a concomitant decrease in overall yield. To mitigate yield losses associated with intermediate purification, one‐pot ligation strategies, in which multiple ligation reactions are performed without isolation of intermediates, have been explored [39, 40]. A variety of one‐pot ligation approaches have been successfully applied to the chemical synthesis of ubiquitinated proteins [41, 42, 43, 44, 45].

In this study, we prepared homogeneous, sequence‐native PAF15Ub2 by combining an optimized hydrazide‐based convergent synthetic strategy [46] with one‐pot ligation for the synthesis of the N‐terminal region of PAF15Ub2 containing two monoubiquitin moieties. The full‐length and N‐terminal segment of synthetic PAF15Ub2 enabled us to demonstrate that the methyltransferase activity of DNMT1 is induced through the interaction between PAF15Ub2 and the RFTS domain. Furthermore, cryo‐EM analysis revealed that DNMT1 adopts an active conformation upon binding PAF15Ub2 and hemimethylated DNA, in a manner similar to that observed for H3Ub2.

## 2 Results and Discussion

### 2.1 Total Chemical Synthesis of PAF15Ub2

Previous studies have highlighted the difficulty in preparing PAF15Ub2 through recombinant expression followed by enzymatic ubiquitination. Although full‐length PAF15 can be expressed in *E. coli*, its purification requires multiple chromatographic steps to separate the desired full‐length protein from truncated species generated during the expression process [47]. Furthermore, in vitro ubiquitination of PAF15 using the E2 enzyme UBE2D and the E3 ligase UHRF1 produces not only the di‐ubiquitinated protein but also mono‐ and polyubiquitinated species [20, 48]. Consistent with these reports, we found that in vitro ubiquitination of the truncated segment PAF15(2–71) by UHRF1 yielded heterogeneous products consisting of mono‐, di‐, and polyubiquitinated species because of low site selectivity and incomplete conversion (Figure S1). These findings underscore the inherent challenges of enzymatic PAF15Ub2 preparation and motivated us to pursue the chemical synthesis of PAF15Ub2.

PAF15Ub2 contains two ubiquitin (76 aa) moieties conjugated via their C‐termini to side‐chain amino groups of K15 and K24 of PAF15 (110 aa), with a total length of 262 aa. Toward the total chemical synthesis of PAF15Ub2, we planned to divide the protein into 6 peptide segments (Figure 2A), two of which are identical Ub(1–45) segments (peptide **1**). Peptides **2** and **3** are branched peptides, each bearing Ub(46–76) on the lysine side chains of PAF(2‐18) and PAF(19‐31), respectively. All segments were designed to comprise fewer than 60 residues to allow manageable preparation by single‐shot SPPS and the segments were assembled by NCL [33]. One cysteine residue and three alanine residues were selected as the ligation junctions (i.e., Ub(F45–A46), PAF(A18–A19), PAF(S31–A32), and PAF(V53‐C54)), as cysteine can be converted to Ala by free‐radical desulfurization [34] after NCL. Since PAF15Ub2 possesses two native cysteine residues (C54 and C99) in the C‐terminal region of PAF15, in our first attempt, we employed a sequential N‐to‐C assembly and performed desulfurization prior to the final ligation step to keep the native cysteine residues intact (Figure S2A). However, the overall yield was only 0.07%, and the isolated PAF15Ub2 was not suitable for subsequent experiments due to its low purity (Figure S2B,C). Two factors likely account for this low yield and purity. First, in the final ligation step, the ligation junction involved a valine–cysteine site, where the inherently slow ligation kinetics [49] could allow competing hydrolysis of the thioester to predominate. Second, in the final ligation step, the small relative difference in size between the ligation reactant and product resulted in nearly identical HPLC retention times, hampering both reaction monitoring and product purification (Figure S2B). To address these limitations, we devised an alternative synthetic scheme, in which peptide segments were assembled in a convergent manner (Figure 2B). This approach avoids employing the valine–cysteine ligation junction in the final step and was expected to give a larger difference in retention time between the reactants and the product, facilitating both reaction monitoring and product isolation. Importantly, this convergent strategy is accelerated by the incorporation of selenocysteine at the final ligation junction, which can be selectively converted to alanine without affecting native cysteine [35].

**FIGURE 2:**
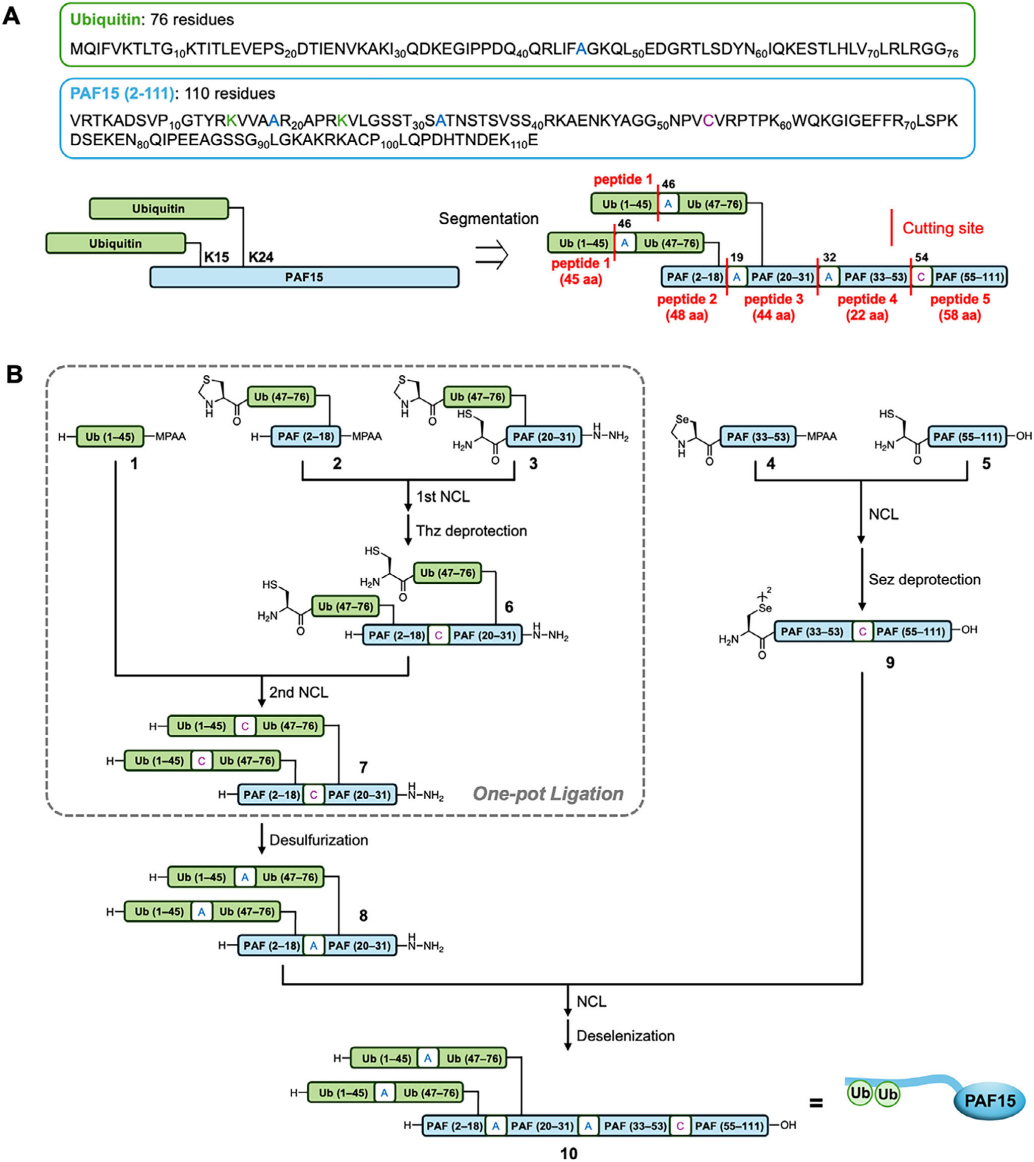
Chemical synthesis of dualmonoubiquitinated PAF15 (**10**). (A) Amino‐acid sequences of ubiquitin and PAF15 and their segmentation. (B) 2nd‐generation synthetic scheme of di‐monoubiquitinated PAF15 (**10**).

Each peptide segment used in this convergent synthetic scheme was synthesized by Fmoc SPPS. Peptide **1** was synthesized as a C‐terminal peptide hydrazide in SPPS and after TFA cleavage, the crude peptide was converted in solution to the 4‐mercaptophenylacetic acid (MPAA) thioester by NaNO_2_‐mediated activation [50]. Following preparative HPLC purification, peptide thioester **1** was obtained in 3.8% overall yield as a lyophilized powder (Figure S3), which could be used directly in the one‐pot ligation without further activation. Branched peptide **2** was synthesized using Fmoc‐Lys(Alloc)‐OH at the ubiquitination site: the main chain was elongated first, capped with Boc‐Val‐OH at its N terminus and, after Alloc removal, the branch was then synthesized from the side‐chain amino group of Lys. A thiazolidine (Thz) moiety, a protected form of N‐terminal cysteine that prevents undesired side‐reactions such as cyclization and oligomerization of this peptide, was introduced at the N terminus of the branch. Of note, Thz was the first N‐terminal cysteine protecting group applied for one‐pot NCL [51]. For the C‐terminal thioesterification, acetylacetone‐mediated activation of peptide hydrazide [52] was employed because NaNO_2_‐mediated activation is known to be incompatible with Thz‐containing peptides [46]. Preparative HPLC purification and lyophilization gave the branched peptide MPAA thioester **2** in 3.4% overall yield (Figure S4). The other branched peptide **3** was prepared by a strategy analogous to that used for peptide **2**, except that Alloc‐Lys(Fmoc)‐OH was employed at the ubiquitination site, allowing branch assembly prior to elongation of the main chain. Peptide **3** requires an unprotected N‐terminal cysteine on the main chain for the first NCL, and was therefore purified and isolated as the hydrazide rather than as a thioester, affording peptide **3** in 7.6% overall yield (Figure S5). The hydrazide was converted to the corresponding thioester prior to the final ligation. Peptide **4** was synthesized with an N‐terminal selenazolidine, the protected form of selenocysteine [53], and C‐terminal hydrazide. As in the case of peptide **2**, the crude peptide was converted to the MPAA thioester by acetylacetone‐mediated activation. Following preparative HPLC purification, peptide thioester **4** was obtained in 5.1% overall yield (Figure S6). C‐terminal peptide **5** with N‐terminal naked cysteine and C‐terminal carboxylic acid was synthesized via standard Fmoc SPPS conditions and obtained in 14% isolated yield (Figure S7).

For the assembly of the N‐terminal region of PAF15Ub2, one‐pot ligation of peptides **1**, **2** and **3** was conducted to generate dual‐monoubiquitinated peptide segment **7** (Figure 3A). The first NCL between peptides **2** and **3** proceeded cleanly at pH 6.5 and was completed within 1 h to afford ligated peptide **2–3** as a single major peak in the analytical HPLC profile. For deprotection of two Thz groups in both branches, we utilized our previously reported Thz deprotection reagent, 2‐amino‐5‐methoxy‐*N*,*N*‐dimethylbenzohydrazide (AMDBH), which is suitable for one‐pot ligation of three or more peptide segments because of the lower nucleophilicity than that of the conventional reagent methoxyamine, leading to only negligible degradation of peptide thioesters [44]. To the reaction mixture, AMDBH was added and the pH was adjusted to 4 to initiate the Thz ring‐opening reaction. ESI–MS analysis after 40 h confirmed the conversion of the Thz groups at the N‐termini of both branches into free cysteines, affording peptide **6** (Figure S8). After readjustment of the pH to 6.5, peptide **1** was subsequently added to the mixture to conduct the second NCL, completing full‐length ubiquitin on both branches. Peptide **1** was used in a slightly substoichiometric amount (1.9 equiv. relative to the two branches), since we had found in preliminary experiments that its hydrolyzed byproduct co‐elutes with peptide **7** on HPLC, which would complicate purification. After 3 h, the reaction mixture was purified by HPLC and peptide **7** was isolated in 38% yield over three steps (Figure S9). Desulfurization was then performed to convert the cysteine residues at the ligation junctions to native alanine residues [34]. After 20 h, completion of desulfurization was verified by LC–MS analysis, and peptide **8** was purified by HPLC and obtained in 49% isolated yield (Figure 3B and Figure S10).

**FIGURE 3:**
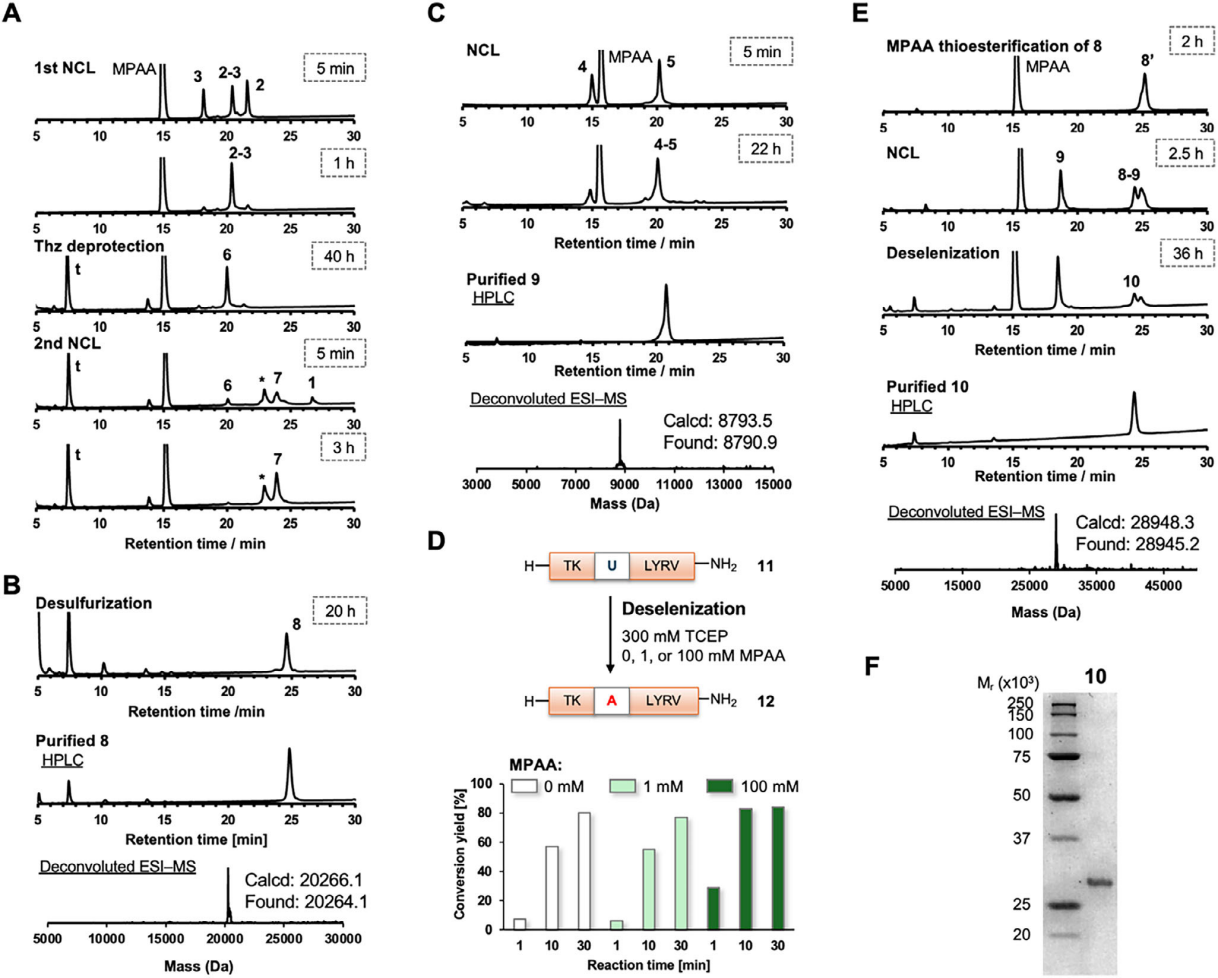
Reaction monitoring of PAF15Ub2 synthesis and characterization of the purified peptides. (A) One‐pot ligation of **1**‐**3**. **t** denotes the formaldehyde‐trapped form of AMDBH. * denotes the mono‐ligated intermediate. HPLC gradient: acetonitrile 15%–45% over 30 min. (B) Desulfurization reaction of **7**. HPLC gradient: acetonitrile 15%–45% over 30 min. (C) Ligation of **4** and **5** and Sez deprotection. HPLC gradient: acetonitrile 15%–40% over 30 min. (D) Deselenization of model peptide **11** at varying MPAA concentrations. (E) Ligation of **8** and **9** and deselenization. **8’** is a thioester of **8**. HPLC gradient: acetonitrile 15%–45% over 30 min. (F) SDS‐PAGE analysis of **10**. HPLC column used in all analyses: Jupiter 5C4 300Å (4.6 mm i.d. × 250 mm) (Phenomenex). Deconvoluted ESI–MS spectra were generated using UniDec [54].

For the assembly of the C‐terminal segment, peptide ligation between peptides **4** and **5** was carried out (Figure 3C). Importantly, the NCL reaction mixture contained ascorbic acid, which suppresses TCEP‐mediated deselenization of selenocysteine or selenazolidine [55, 56]. Completion of NCL was confirmed after 22 h and ligated peptide **4–5** was observed as a main peak in an analytical HPLC profile. Methoxyamine was then added to the mixture and the pH was adjusted to 3 to convert the N‐terminal selenazolidine to selenocysteine [53]. After HPLC purification, peptide **9** was obtained in 65% isolated yield (Figure S11).

The final step required a selenocysteine‐mediated NCL between peptides **8** and **9** followed by deselenization, and we aimed to perform these two reactions in one pot to maximize the isolated yield of the final product. The key insight here is that NCL mediated by the nucleophilic attack of an N‐terminal selenocysteine proceeds in the presence of an excess of MPAA even without TCEP, since MPAA partially reduces the diselenide to generate the reactive selenol [35]. MPAA, however, is an efficient radical scavenger that inhibits radical‐mediated desulfurization of cysteine [57, 58], and is thus also thought to inhibit the deselenization of selenocysteine [35, 59]. Nevertheless, deselenization of selenoproline has been achieved after peptide ligation in one pot without removing MPAA [60], suggesting that the selenocysteine deselenization may tolerate MPAA. We therefore examined, using a selenocysteine‐containing model peptide **11**, whether deselenization would proceed in the presence of MPAA (Figure 3D and Figure S15). Unexpectedly, deselenization of peptide **11** proceeded rapidly even in the presence of 100 equivalents of MPAA to afford the Ala‐containing peptide **12**, and reached completion slightly faster than in the presence of 1 equivalent of MPAA or in the absence of MPAA. This tolerance to MPAA can be rationalized by the comparable stabilities of the aryl thiyl radical derived from MPAA and the alkyl selanyl radical derived from selenocysteine [61, 62]. Since the two radicals are energetically similar, MPAA would not sequester the chain‐carrying selanyl radical, in contrast to radical desulfurization, in which the more reactive alkyl thiyl radical is efficiently scavenged by MPAA. These results suggest that the selenocysteine‐mediated final ligation between peptides **8** and **9** and the subsequent deselenization can be performed in a one‐pot fashion.

In the final ligation step (Figure 3E), the C‐terminal hydrazide of peptide **8** was first converted to the MPAA thioester using acetylacetone. Completion of the thioesterification of peptide **8** to yield peptide **8’** was confirmed by LC–MS analysis after 2 h (Figure S12). Addition of peptide **9** to the reaction mixture initiated the final NCL in the presence of MPAA but in the absence of TCEP and ascorbic acid, affording peptide **8–9** within 2.5 h. After incubation for another 6 h, an excess of TCEP was added to the mixture to selectively convert the selenocysteine residue to alanine via deselenization. After 36 h, completion of deselenization was verified by LC–MS analysis. The target protein PAF15Ub2 (**10**) was isolated in 13% yield for this final ligation–deselenization step, and with high purity as confirmed by HPLC, LC–MS, and SDS‐PAGE (Figure 3E,F and Figure S13). Compared to our first attempt, this second‐generation strategy afforded a more than 30‐fold improvement in the overall isolated yield (from 0.07% to 2.7%), achieved by altering the order of segment assembly and utilizing a one‐pot ligation strategy and deselenization chemistry. This overall yield is reasonable compared to previously reported syntheses of ubiquitinated proteins such as K27‐linked di‐ubiquitinated histone H2A (7.8%) [63] and SUMO‐2 bearing a Lys63‐linked di‐ubiquitin chain (6%) [64].

### 2.2 Functional Validation of Chemically Synthesized PAF15Ub2

Dual‐monoubiquitination at K15 and K24 on PAF15 functions as a signal for recruitment of DNMT1 onto the early replication sites, in which the RFTS domain specifically recognizes the ubiquitinated PAF15 [20]. To test whether the chemically synthesized PAF15Ub2 is capable of binding to the RFTS domain, we conducted an electrophoretic mobility shift assay (EMSA) using the purified recombinant RFTS domain (351–600 aa) (Figure 4A). EMSA under non‐denaturing conditions using polyacrylamide gel electrophoresis showed concentration‐dependent mobility shifts of the RFTS domain with both peptides **8** and **10**, indicating that both synthetic PAF15Ub2 constructs retain the ability to be recognized by the RFTS domain.

**FIGURE 4:**
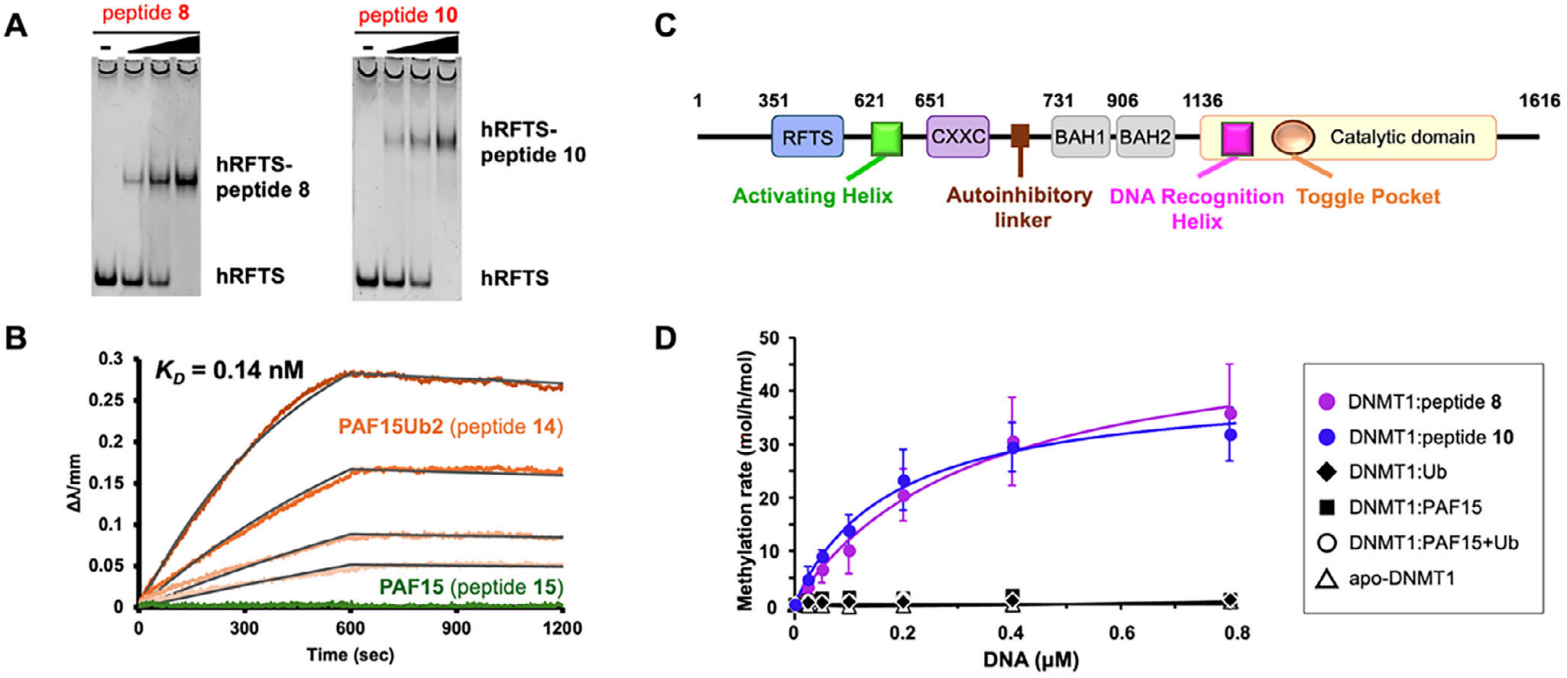
Binding and functional validation of chemically synthesized PAF15Ub2. (A) Binding assay of the synthetic PAF15Ub2 against DNMT1. Electrophoresis mobility shift assay (EMSA) to detect the interaction between hRFTS domain (351‐600 aa) and N‐terminal peptide **8** (left), full‐length peptide **10** (right). (B) Binding analysis by BLI. Biotinylated N‐terminal peptides of PAF15Ub2 and unmodified PAF15 (peptides **14** and **15**, respectively) were immobilized on streptavidin biosensors. For the kinetic analysis of peptide **14**, the hRFTS domain was used at 0.5, 1.0, 2.0, and 4.0 nM (orange); for peptide **15**, the hRFTS domain was used at 8.0 nM (green). (C) Domain structure of DNMT1 with amino acid numbers. (D) In vitro DNA methylation activity of DNMT1. Apo‐DNMT1 (white‐triangle) and DNMT1 in the presence of N‐terminal peptide **8** (magenta‐circle), full‐length peptide **10** (blue‐circle), PAF15 (black‐square), Ub (black‐diamond) and Ub mixed with non‐ubiquitinated PAF15 (white‐circle). At least three independent experiments were performed for estimation of standard deviation (error bars represent ± SD of *n* = 3).

A biolayer interferometry (BLI) assay was then performed to gain further insight into the interaction between the RFTS domain and PAF15Ub2. For this measurement, we synthesized biotinylated peptides corresponding to the N‐terminal regions of PAF15Ub2 (peptide **14**) and of unmodified PAF15 (peptide **15**) (Figures S16–S19). The RFTS domain bound to peptide **14** with a dissociation constant of 0.14 nM but did not bind to non‐ubiquitinated peptide **15** (Figure 4B). Previous studies reported dissociation constants of 1.4 [20] and 29 nM [21], indicating that PAF15Ub2 prepared here binds the RFTS domain with higher affinity. One possible explanation for this difference is the use of the native isopeptide linkage in place of the disulfide linkage employed in the previous PAF15Ub2 analogs.

Next, we examined whether the synthetic PAF15Ub2 relieves the autoinhibition of DNMT1. We performed an in vitro DNA methylation assay using recombinant DNMT1 (351‐1616 aa) [10], which retains both the RFTS and catalytic domains (Figure 4C). Neither ubiquitin nor PAF15 alone, both prepared as recombinant proteins from *E. coli*, nor a simple mixture of the two exhibited any detectable enhancement of DNA methylation activity of DNMT1 (Figure 4D). In clear contrast, the chemically synthesized PAF15Ub2, peptides **8** and **10**, markedly increased the DNA methylation activity. These results indicate that PAF15Ub2 functions as a direct activator of DNMT1, consistent with the reported role of ubiquitinated H3.

### 2.3 Cryo‐EM Structural Analysis of DNMT1 Bound to PAF15Ub2 and Hemimethylated DNA

The autoinhibitory conformation of apo‐DNMT1 mediated by the RFTS domain and the autoinhibitory linker [6, 7] is relieved upon binding to H3Ub2 and hemimethylated DNA [9, 10]. In addition to these inhibitory domains, we previously discovered an activation–inactivation “Toggle Pocket” within the catalytic domain of DNMT1 [10]. In the inactive state, Phe1235, Tyr1240, and Phe1243 of the DNA Recognition Helix occupy the Toggle Pocket. Upon activation triggered by binding to H3Ub2 and hemimethylated DNA, the side chains of Phe631 and Phe632 in the Activating Helix insert into the Toggle Pocket, allowing the DNA Recognition Helix to fully access the DNA substrate [10]. In contrast, the role of PAF15Ub2 in DNMT1 activation remains to be fully elucidated. To reveal whether PAF15Ub2 also changes the structure of DNMT1 to the activating form, cryo‐EM single particle analysis of the DNMT1:PAF15Ub2:hemimethylated DNA ternary complex was conducted. The mixture of DNMT1 (351‐1616 aa):PAF15Ub2 segment **8** was reacted with S‐adenosyl‐L‐methionine (SAM) and hemimethylated DNA (DNA^mCG/fCG^), in which the cytosine to be methylated was replaced by a 5‐fluorocytosine (fC) to form an irreversible covalent intermediate [65]. The ternary complex of DNMT1 bound to PAF15Ub2 and DNA^mCG/fCG^ was purified by size‐exclusion chromatography and used for the cryo‐EM single particle analysis (Figure S20).

The cryo‐EM map of the ternary complex was constructed at 3.25 Å resolution, which allowed us to successfully build the atomic model of DNMT1 (Figures S21 and S22). The overall structure of the ternary complex is almost the same as the previously determined structure of the DNMT1:H3Ub2:DNA^mCG/fCG^ ternary complex with a Cα atom root mean square deviation of 0.66 Å. The cryo‐EM map corresponding to the RFTS domain bound to PAF15Ub2, the CXXC domain and the autoinhibitory linker is fully invisible, indicating dissociation of these moieties from the catalytic domain (Figure 5A). In our previous structure of the DNMT1–H3Ub2 complex, maps both with and without density for the CXXC domain were obtained, and the population lacking visible CXXC domain density was predominant [10], indicating that the positioning of the CXXC domain is intrinsically flexible. In the DNMT1–PAF15Ub2 complex, only maps lacking density for the CXXC domain were obtained. Binding of PAF15Ub2 and DNA^mCG/fCG^ switches the Toggle Pocket to the active state (Figure S23A): the aromatic side chains of Phe631 and Phe632 in the Activating Helix are inserted into the Toggle Pocket of the catalytic domain (Figure 5B). The DNA Recognition Helix adopts a straight conformation that enables binding to cognate DNA. These structural insights indicate that DNMT1 switches to the active conformation upon binding PAF15Ub2 and DNA^mCG/fCG^ in a manner analogous to the H3Ub2‐bound complex (Figure S23B), consistent with the previously proposed model [22].

**FIGURE 5:**
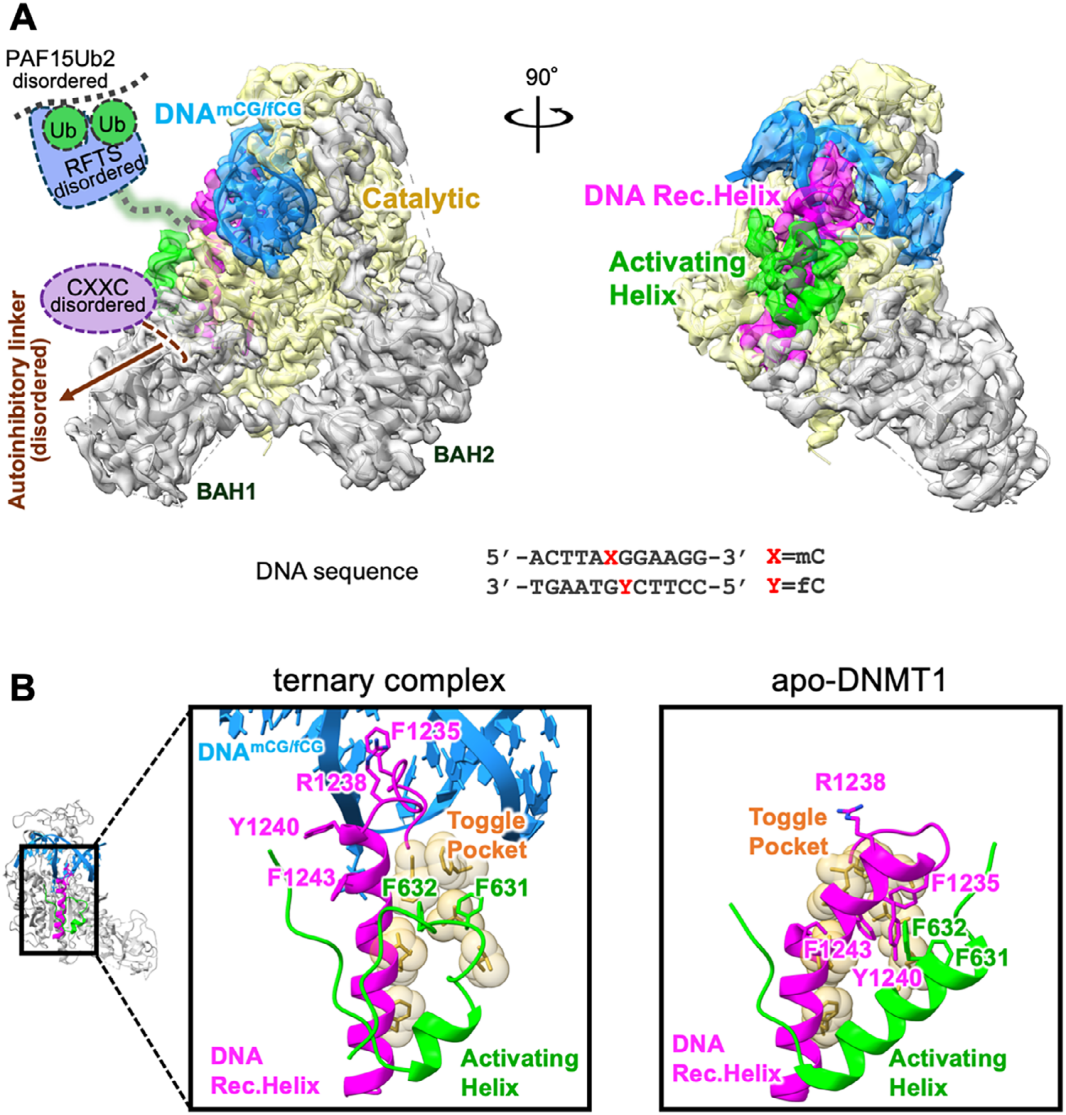
Cryo‐EM single particle analysis of DNMT1:PAF15Ub2:DNA^mCG/fCG^ ternary complex. (A) Cryo‐EM map of the DNMT1:PAF15Ub2:DNA^mCG/fCG^ ternary complex superposed on the cartoon model. Disordered RFTS domain bound toPAF15Ub2, CXXC domain and autoinhibitory linker is shown as dotted lines. (B) Structure around Toggle Pocket (light yellow) of the ternary complex (left panel). Toggle Pocket is shown as a stick with a transparent sphere model. Residues in Activating Helix and DNA Recognition Helix that are involved in binding to the Toggle Pocket are shown as green and magenta stick models, respectively. Structure around Toggle Pocket of apo‐DNMT1 is shown in the right panel (PDB ID: 4WXX).

## 3 Conclusions

In summary, we established a synthetic route to PAF15Ub2 and used the synthetic protein to investigate the mechanism of DNMT1 activation. Our initial sequential peptide ligation strategy failed to afford the desired product in sufficient yield and purity due to the repeated HPLC purification and the poor separation of the final product, respectively. We therefore adopted a convergent synthetic strategy combined with a one‐pot ligation approach, which reduced the number of intermediate purification steps and improved the HPLC resolution between the reactant and product in the final ligation relative to the first‐generation route, thereby improving the overall yield and the purity of the final product. We further demonstrated that the deselenization of selenocysteine to alanine proceeds even in the presence of an excess of MPAA, which allowed the final ligation and deselenization to be performed in one pot, further reducing the number of purification steps in the synthesis of PAF15Ub2. Using the chemically synthesized PAF15Ub2, cryo‐EM analysis of the DNMT1:PAF15Ub2:DNA^mCG/fCG^ ternary complex was successfully performed and revealed that PAF15Ub2 and DNA^mCG/fCG^ are associated with the active conformation of DNMT1, similarly to H3Ub2. Together with the biochemical evidence that the RFTS domain binds the N‐terminal dual‐monoubiquitinated region of PAF15Ub2 and that the methyltransferase activity of DNMT1 is directly enhanced by PAF15Ub2, our structural findings support a model in which PAF15Ub2 activates DNMT1 through a mechanism analogous to that of H3Ub2 (Figure 6). In the apo state, the RFTS domain, CXXC domain, and autoinhibitory linker cover the catalytic domain. In the ternary complex, by contrast, these elements are released from the catalytic domain, and the catalytic site is occupied by DNA^mCG/fCG^.

**FIGURE 6:**
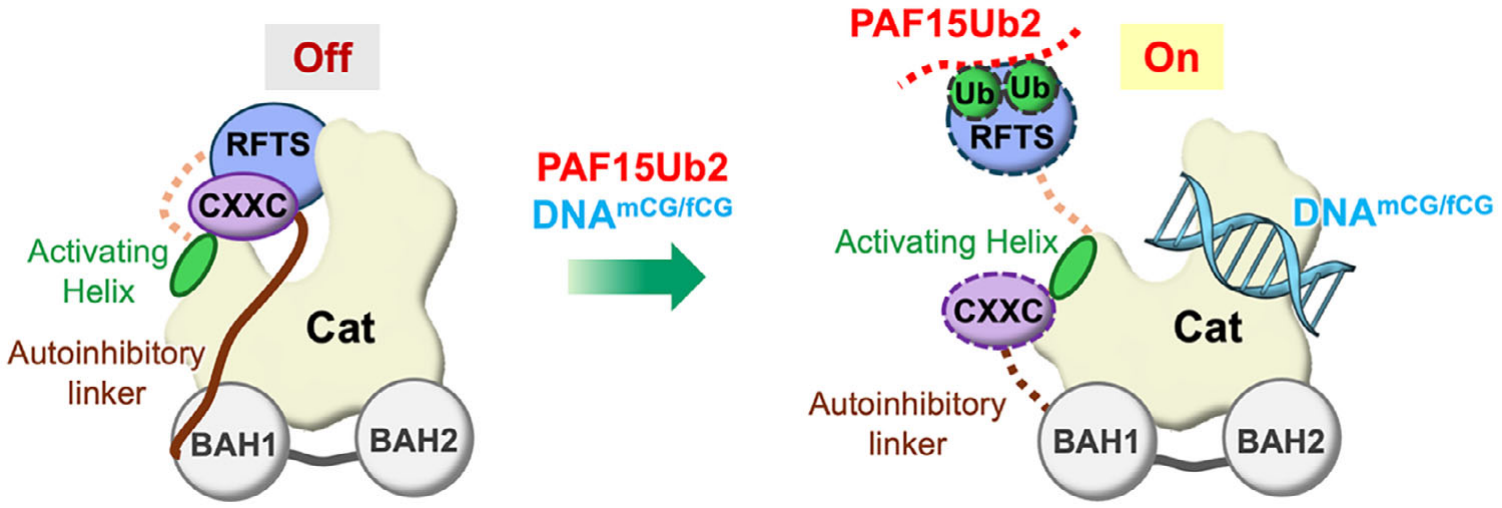
Schematic representation of the DNMT1 activation mechanism mediated by PAF15Ub2. Regions not resolved in the cryo‐EM map are shown as dotted lines.

While the structural basis for recognition of H3Ub2 by the RFTS domain has been established [9], how PAF15Ub2 is recognized remains unclear. Given that the N‐terminal dual‐monoubiquitinated moieties of PAF15 and H3 differ in terms of their primary sequences and the spacing between the ubiquitinated Lys residues, PAF15Ub2 could be recognized by the RFTS domain through a distinct binding mode. Furthermore, the synthetic full‐length PAF15Ub2 developed in this study may facilitate cryo‐EM analysis of the DNMT1:PAF15Ub2:hemimethylated DNA:PCNA quaternary complex, which has been challenging due to the instability of the complex in previous attempts [22]. In cases where the complex lacks sufficient stability for structural analysis, chemical protein synthesis would enable the site‐specific installation of a covalent crosslinker in PAF15Ub2 to tether PCNA, thereby stabilizing the complex assembly and allowing structural characterization.

## Author Contributions

**Sae Suzuki**: funding acquisition, writing – original draft, writing – review and editing, data curation, visualization, investigation, validation, methodology. **Yuya Takahashi**: writing – original draft, visualization, data curation, validation, investigation. **Amika Kikuchi**: validation, visualization, writing – review and editing, writing – original draft, data curation. **Satomi Kori**: validation, visualization, investigation. **Fumika Yoshitomi**: investigation, validation. **Hiroshi Murakami**: supervision, funding acquisition. **Kyohei Arita**: conceptualization, funding acquisition, methodology, supervision, project administration, resources, writing – original draft, writing – review and editing. **Gosuke Hayashi**: writing – original draft, writing – review and editing, conceptualization, funding acquisition, supervision, project administration, resources, methodology.

## Conflicts of Interest

The authors declare no conflicts of interest.

## Supporting information




**Supporting File**: advs78636‐sup‐0001‐SuppMat.docx.

## Data Availability

The cryo‐EM density map has been deposited in the Electron Microscopy Data Bank (EMDB, www.ebi.ac.uk/pdbe/emdb/) under accession code EMD‐38573, and the atomic coordinates have been deposited in the PDB (www.rcsb.org) under accession code 8XQC. All data needed to evaluate the conclusions in the paper are present in the paper and/or the Supplementary Information. PDB 7XI9 and 4WXX were used for this study.
