# Supplementary material for: Activation of DNMT1 by Chemically Synthesized Dual‐Monoubiquitinated PAF15 Protein
Source: Adv Sci (Weinh). 2026 Nov 10:e78636. Online ahead of print. doi: 10.1002/advs.78636 (PMC13752237; doi:10.1002/advs.78636)
Supplement: Supplementary file 1 [file ADVS-9999-e78636-s001.docx]

**Activation of DNMT1 by Chemically Synthesized Dual monoubiquitinated PAF15 Protein**

Sae Suzuki^a^, Yuya Takahashi^a^, Amika Kikuchi^b^, Satomi Kori^b^, Fumika Yoshitomi^a^, Hiroshi Murakami^a,c,d^*, Kyohei Arita^b,e^*, Gosuke Hayashi^a,f,g^*

^a^Department of Biomolecular Engineering, Graduate School of Engineering, Nagoya University, Furo-cho, Chikusa-ku Nagoya 464-8603, Japan

^b^Graduate School of Medical Life Science, Yokohama City University, Tsurumi-ku, Yokohama, Kanagawa 230-0045, Japan

^c^Institute of Nano-Life Systems, Institutes of Innovation for Future Society Nagoya University, Furo-cho, Chikusa-ku Nagoya 464-8603, Japan

^d^Research Institute for Quantum and Chemical Innovation, Institutes of Innovation for Future Society Nagoya University, Furo-cho, Chikusa-ku Nagoya 464-8603, Japan

^e^Institute of Advanced Medical Sciences, Tokushima University, 3-18-15 Kuramoto-cho, Tokushima 770-8503, Japan

^f^Institute for Life and Medical Sciences, Kyoto University, 53 Shogoin-Kawahara-cho, Sakyo-ku, Kyoto 606-8507, Japan

^g^Department of Chemical Science and Engineering, Graduate School of Engineering, Kyoto University, Kyotodaigaku-Katsura, Nishikyo-ku, Kyoto 615-8510, Japan

**Table of Contents**

1. **Experimental Section** ...................................................................................................................... S3
   1.1. General ........................................................................................................................................ S3
   1.2. Synthesis of Boc-selenazolidine-OH (**S1**) .................................................................................... S3
   1.3. Peptide synthesis ......................................................................................................................... S4

1.3.1. General protocol of peptide synthesis ........................................................................................ S4

1.3.2. Peptide preparation for diubiquitinated PAF15 (**1**,**2**,**3**,**4**,**5**) .…................................................... S4

1.3.3. Preparation of model peptide (**11**) .……………………………………….………………………… S7

1.3.4. Preparation of biotinylated peptides (**13**, **15**)……………….…………………………….………… S7

1.4. Chemical synthesis of PAF15Ub2 .................................................................................................. S8

1.4.1. One-pot ligation of **1**, **2** and **3** (**7**) ............................................................................................... S8

1.4.2. Desulfurization (**8**) ..................................................................................................................... S9

1.4.3. Ligation of **4** and **5** and Sez deprotection (**9**) …......................................................................... S9

1.4.4. Ligation of **8** and **9** and deselenization (**10**) ............................................................................. S10

1.5. Deselenization of model peptide **11** .………………………………….……………….……………… S10

1.6. Electrophoretic mobility shift assay (EMSA) ................................................................................. S10

1.7. Preparation of biotinylated N-terminal segment of PAF15Ub2 (**14**) ………………………….…… S11

1.8. Affinity measurement of PAF15Ub2 against hRFTS domain by BLI ………………………………. S11

1.9. *in vitro* DNA methylation assay .................................................................................................... S11

1.10. Cryo-EM analysis ...................................................................................................................... S12

1.10.1. Preparation of ternary complex for cryo-EM single particle analysis ................................... S12

1.10.2.　Data collection .................................................................................................................... S12

1.10.3.　Data processing ……………………………………………………….…….…………………… S12

1.10.4.　Model building and validation …………………………….….…….….….……………………. S13

2. **Supporting Figures** ........................................................................................................................ S15
3. **NMR spectra** ................................................................................................................................... S31
1. **Experimental Section**

**1.1 General**

All Fmoc- or Boc-protected amino acids were purchased from Watanabe Chemical, Novabiochem, or Peptide Institute, Inc. Other chemicals were purchased from Sigma-Aldrich, Wako chemicals, Tokyo Chemical Industry, or other commercial suppliers. All solvents and reagents were used without further purification.

NMR spectra were recorded with AVANCE III HD 300MHz-1 (Bruker). ESI–MS analysis of peptides was performed by InfinityLab LC/MSD G6125B (Agilent). MALDI-TOF MS measurements were performed by autoflex max (Bruker). Preparative HPLC purifications of peptides were carried out using PU-2080 Plus Intelligent HPLC Pump (JASCO) and MD-2018 Plus Photodiode Array Detector (JASCO) monitored at 195–650 nm with one of the following columns: 5C18 AR-II column (20 mm i.d. × 250 mm) (Nacalai), Protein-R (20 mm i.d. × 250 mm) (Nacalai) or Jupiter 5C4 300Å Column (20 mm i.d. × 250 mm) (Phenomenex). A binary mixture of A (water containing 0.1% trifluoroacetic acid (TFA)) and B (acetonitrile containing 0.1% TFA) was used as the mobile phase at a flow rate of 7.0 or 7.5 mL/min) under linear gradient conditions. For analytical HPLC measurements of peptides, 5C18 AR-II column (4.6 mm i.d. × 250 mm) (Nacalai), Protein-R column (4.6 mm i.d. × 250 mm) (Nacalai), or Jupiter 5C4 300Å Column (4.6 mm i.d. × 250 mm) (Phenomenex) was used with a binary mixture of A and B as a mobile phase (flow rate = 1.0 mL/min) in a linear gradient as described at each figure or protocol. All HPLC traces shown in this paper are monitored at 220 nm. All heating protocols were conducted in a cool incubator ICI-100 (AS ONE) or COOL-INCUBATOR HCRCS2V75W-A0602 (IKUTA sangyo).

**1.2 Synthesis of Boc-selenazolidine-OH (S1)**

Scheme S1. Synthetic scheme of Boc-Selenazolidine-OH

Boc-selenazolidine-OH was synthesized following the previously reported procedure [53], with minor modifications. *L*-Selenocystine (167 mg, 500 µmol) was dissolved in 7.75 mL of degassed 50 mM NaOH aq. and 2.25 mL of EtOH. NaBH_4_ (66.2 mg, 1.75 mmol) was added to the reaction mixture portionwise under an argon atmosphere. The reaction solution was stirred for 12 h on ice. Stirring was continued until the solution became colorless. The pH was adjusted to 4–5 with 6 M HCl. Then, 37% formaldehyde aq. (239 µL, 6.5 equiv.) was added dropwise and the reaction mixture was stirred for 2 h under an argon atmosphere. The pH was adjusted to 8 with K_2_CO_3_, then Boc_2_O (437 mg, 2.00 mmol) dissolved in 1.25 mL of dioxane was added to the reaction. The reaction was stirred for 1 h and another 2.00 mmol of Boc_2_O was added. After stirring for 2.5 h, pH was adjusted to 9 with K_2_CO_3_ and the reaction was stirred for 19 h. The crude mixture was diluted with water and washed with Et_2_O. The aqueous layer was acidified to pH 3–4 with citric acid aq. and extracted with EtOAc (2 times). The combined organic layers were washed with citric acid aq. and brine, dried over MgSO_4_, and concentrated in vacuo. Column chromatography was performed (CHCl_3_/MeOH = 100/0 to 90/10) and the product (**S1**) was obtained as a low-melting, pale yellow solid in 70% yield (196 mg, 699 µmol). ^1^H NMR (300 MHz, CDCl_3_): δ = 7.0~8.8 (brs, 1H, COOH), 5.36 (brs, 0.6H, NCH_2_Se), 5.13 (brs, 0.4H, NCH_2_Se), 4.97-4.72 (m, 1H, NCH_2_Se), 4.60-4.39 (m, 1H, CαH), 3.47-3.22 (m, 2H, CβH_2_), 1.48 (s, 9H, CMe_3_).

**1.3 Peptide Synthesis**

**1.3.1 General protocol of peptide synthesis**

Automated solid-phase peptide synthesis was performed using a MultiPep RS (Intavis) for parallel synthesis, Liberty Blue (CEM) or Initiator+ Alstra (Biotage) via the standard Fmoc-SPPS protocol. For Liberty Blue, amino acids protected by the 9-fluorenylmethoxycarbonyl (Fmoc) group were coupled with *N,N*’-diisopropylcarbodiimide (DIC) and ethyl (hydroxyimino)cyanoacetate (OxymaPure). For other peptide synthesizers, Fmoc-protected amino acids were coupled with *O*-(1*H*-Benzotriazol-1-yl)-*N,N,N’,N'*-tetramethyluronium hexafluorophosphate (HBTU) as an activator and *N,N*-diisopropylethylamine (DIEA) as a base. Manual solid-phase peptide synthesis was performed as follows. Coupling of Fmoc-Asp(OtBu)-(Dmb)Gly-OH: the Fmoc-protected amino acid (3 equiv.) was activated with HATU (2.9 equiv.) and DIEA (6 equiv.) in DMF and added to the resin (coupling time: 60 min at room temperature), coupling of Fmoc-Lys(biotin)-OH: the Fmoc-protected amino acid (4 equiv.) was activated with HATU (3.8 equiv.) and DIEA (8 equiv.) in the mixture of DMF and NMP and added to the resin (coupling time: 5 min at 75 °C), coupling of Boc-Selenazolidine-OH: the Boc-protected amino acid (3 equiv.) was activated with DIC (3 equiv.) and 7-Aza-1-hydroxybenzotriazole (HOAt) (3 equiv.) in DMF and transferred to the resin (coupling time: 60 min at room temperature), coupling of Fmoc-Sec(PMB)-OH: the Fmoc-protected amino acid (3 equiv.) was activated with DIC (3 equiv.) and OxymaPure (3 equiv.) in DMF and transferred to the resin (coupling time: 3 h at room temperature), couplings following the introduction of Sec: the Fmoc-protected amino acid (4 equiv.) was activated with DIC (4 equiv.) and OxymaPure (4 equiv.) in DMF and added to the resin (coupling time: 2 h at room temperature). For Fmoc deprotection, the resin was treated with 20% piperidine in DMF for 5 + 10 min. The isolated yields were calculated based on the molecular weight of the peptide as the TFA salt form, accounting for TFA counterions at the N-terminal amine and the side chains of Arg, Lys, and His.

**1.3.2 Peptide preparation for diubiquitinated PAF15 (1,2,3,4,5)**

**Peptide synthesis of Ub(1-45)-SR (1)**

Ub(1–45)-SR (**1**) was prepared with Cl-Trt(2-Cl)-Resin (Watanabe chemical, 1.0–1.8 mmol/g, 50 μmol scale). After swelling the resin in 50% DMF/DCM for 30 min, Fmoc-NHNH_2_ (1.1 equiv.) and DIEA (2.2 equiv.) in DMF were added to the resin, and the mixture was stirred at room temperature for 12 h. To cap the unreacted trityl group, MeOH was added and the mixture was agitated at room temperature for 10 min. This peptide was elongated by Liberty Blue (CEM). To suppress aspartimide formation, Fmoc-Asp(OMpe)-OH was used for the Asp_21_, Asp_32_, and Asp_39_ residues instead of standard Fmoc-Asp(OtBu)-OH. The resin was washed with DMF and DCM, dried in vacuo and treated with a cleavage cocktail (95% TFA, 2.5% triisopropylsilane (TIPS), 2.5% H_2_O) for global deprotection and release of the peptide. The mixture was stirred at room temperature for 2 h. Then, the solution was filtered to remove the resin, and ten volumes of cold diethyl ether were added to the filtrate to precipitate the peptide. This suspension was vortexed and centrifuged at 10,000 rpm at 4 °C for 10 min. The supernatant was removed by decantation. Obtained pellet was washed twice with diethyl ether. The thioesterification via NaNO_2_ activation was performed according to a previous report [50]. The crude peptide was dissolved in buffer (8 M Gn·HCl, 0.2 M NaH_2_PO_4_ (pH 3.0), 10 mL). The solution was cooled to -15 °C and 1 M NaNO_2_ aq. was added (10 equiv.). The mixture was stirred at -15 °C for 15 min, and then 4-mercaptophenylacetic acid (MPAA) (40 equiv.) was added to the reaction mixture. The pH was adjusted to 5.7 with 6 N NaOH. The peptide solution was diluted with water/acetonitrile containing 0.1% TFA and purified by semi-preparative RP-HPLC. After lyophilization, peptide **1** was obtained as a white solid (11.5 mg, 1.9 μmol, 3.8%). HPLC profile and mass data after purification are shown in Figure S3.

**Peptide synthesis of PAF15(2–18)-[Thz-Ub(47–76)]-SR (2)**

PAF15(2–18)-[Thz-Ub(47–76)]-SR (**2**) was prepared with Cl-Trt(2-Cl)-Resin (Watanabe chemical, 1.0–1.8 mmol/g, 200 μmol scale). After swelling the resin in 50% DMF/DCM for 30 min, Fmoc-NHNH_2_ (1.1 equiv.) and DIEA (2.2 equiv.) in DMF were added to the resin, and the mixture was stirred at room temperature for 12 h. To cap the unreacted trityl group, MeOH was added and the mixture was agitated at room temperature for 10 min. This peptide was elongated by Initiator+ Alstra (Biotage). Fmoc-Lys(Alloc)-OH was used for the Lys_15_ residue of PAF15(2–18). After the coupling of N-terminal Boc-Val-OH for Val_2_ of PAF15(2–18), to remove the Alloc group on Lys_15_, Pd(PPh_3_)_4_ (0.25 equiv.) and PhSiH_3_ (20 equiv.) in degassed DCM were added to the resin and the mixture was stirred for 20 min under an argon atmosphere. This procedure was conducted twice. Then, the branched sequence of Thz-Ub(47–76) was elongated using Initiator+ Alstra (Biotage) with a double coupling protocol. For the Asp_51_-Gly_52_ sequence of Thz-Ub(47–76), Fmoc-Asp(OtBu)-(Dmb)Gly-OH was employed to prevent aspartimide formation. The resin was washed with DMF and DCM, dried in vacuo and treated with a cleavage cocktail (95% TFA, 2.5% TIPS, 2.5% H_2_O) for global deprotection and release of the peptide. The mixture was stirred at room temperature for 2 h. Then, the solution was filtered to remove the resin, and ten volumes of cold diethyl ether were added to the filtrate to precipitate the peptide. This suspension was vortexed and centrifuged at 10,000 rpm at 4 °C for 10 min. The supernatant was removed by decantation. Obtained pellet was washed twice with diethyl ether. The thioesterification via acetylacetone treatment was performed according to a previous report [52]. Briefly, the crude peptide was dissolved in buffer (8 M Gn·HCl, 0.2 M NaH_2_PO_4_ (pH 3.0), 7 mL). MPAA (5 equiv.) and 1 M acetylacetone (2.5 equiv.) in H_2_O were added to the peptide solution and the reaction mixture was stirred at 37 °C for 2 h. Then, the solution was diluted with water/acetonitrile containing 0.1% TFA and purified by semi-preparative RP-HPLC. After lyophilization, peptide **2** was obtained as a white solid (46.6 mg, 6.9 μmol, 3.4%). HPLC profile and mass data after purification are shown in Figure S4.

**Peptide synthesis of Cys-PAF15(19–31)-[Thz-Ub(47–76)]-NHNH_2_ (3)**

Cys-PAF15(19–31)-[Thz-Ub(47–76)]-NHNH_2_ (**3**) was prepared with Cl-Trt(2-Cl)-Resin (Watanabe chemical, 1.0–1.8 mmol/g, 50 μmol scale). After swelling the resin in 50% DMF/DCM for 30 min, Fmoc-NHNH_2_ (1.1 equiv.) and DIEA (2.2 equiv.) in DMF were added to the resin, and the mixture was stirred at room temperature for 12 h. To cap the unreacted trityl group, MeOH was added and the mixture was agitated at room temperature for 10 min. This peptide was elongated by Initiator+ Alstra (Biotage). Alloc-Lys(Fmoc)-OH was used for the Lys_24_ residue of PAF15 (19–31). For the Asp_51_-Gly_52_ sequence of Thz-Ub(47–76), Fmoc-Asp(OtBu)-(Dmb)Gly-OH was employed to prevent the aspartimide formation. After the coupling of N-terminal Boc-Thz-OH for Thz_46_ of the branch sequence, to remove the Alloc group on Lys_24_, Pd(PPh_3_)_4_ (0.25 equiv.) and PhSiH_3_ (20 equiv.) in degassed DCM were added to the resin and the mixture was stirred for 20 min under an argon atmosphere. This procedure was conducted twice. Then, the main-chain sequence of Cys-PAF15(20–23) was elongated using Initiator+ Alstra (Biotage) with a double coupling protocol. The resin was washed with DMF and DCM, dried in vacuo and treated with a cleavage cocktail (90% TFA, 3% 1,2-ethanedithiol, 2% anisole, 5% thioanisole) for global deprotection and release of the peptide. The mixture was stirred at room temperature for 2 h. Then, the solution was filtered to remove the resin, and ten volumes of cold diethyl ether were added to the filtrate to precipitate the peptide. This suspension was vortexed and centrifuged at 10,000 rpm at 4 °C for 10 min. The supernatant was removed by decantation. Obtained pellet was washed twice with diethyl ether. The crude peptide was dissolved in water/acetonitrile containing 0.1% TFA and purified by semi-preparative RP-HPLC. After lyophilization, peptide **3** was obtained as a white solid (27.6 mg, 3.8 μmol, 7.6%). HPLC profile and mass data after purification are shown in Figure S5.

**Peptide synthesis of Sez-PAF15(33–53)-SR (4)**

An N-terminal selenazolidine (Sez) peptide, Sez-PAF15(33–53)-SR (**4**) was prepared with Cl-Trt(2-Cl)-Resin (Watanabe chemical, 1.0–1.8 mmol/g, 50 μmol scale). After swelling the resin in 50% DMF/DCM for 30 min, Fmoc-NHNH_2_ (1.1 equiv.) and DIEA (2.2 equiv.) in DMF were added to the resin, and the mixture was stirred at room temperature for 12 h. To cap the unreacted trityl group, MeOH was added and the mixture was agitated at room temperature for 10 min. This peptide was elongated by Liberty Blue (CEM) except for manual coupling of Boc-Sez-OH (**S1**). The resin was washed with DMF and DCM, dried in vacuo and treated with a cleavage cocktail (95% TFA, 2.5% TIPS, 2.5% H_2_O) for global deprotection and release of the peptide. The mixture was stirred at room temperature for 2 h. Then, the solution was filtered to remove the resin, and ten volumes of cold diethyl ether were added to the filtrate to precipitate the peptide. This suspension was vortexed and centrifuged at 10,000 rpm at 4 °C for 10 min. The supernatant was removed by decantation. Obtained pellet was washed twice with diethyl ether. The crude peptide was dissolved in buffer (8 M Gn·HCl, 0.2 M NaH_2_PO_4_ (pH 3.0), 2.5 mL). MPAA (7.5 equiv.) and 500 mM acetylacetone in H_2_O (2.5 equiv.) were added to the reaction mixture and the reaction mixture was stirred at 37 °C for 2 h. The peptide solution was diluted with water/acetonitrile containing 0.1% TFA and purified by semi-preparative RP-HPLC. After lyophilization, peptide **4** was obtained as a white solid (7.5 mg, 2.6 μmol, 5.1%). HPLC profile and mass data after purification are shown in Figure S6.

**Peptide synthesis of Cys-PAF15(55–111)-OH (5)**

Cys-PAF15(55–111)-OH (**5**) was prepared with Fmoc-Glu(tBu)-Alko-PEG Resin (Watanabe chemical, 0.23 mmol/g, 50 μmol scale). This peptide was elongated by Liberty Blue (CEM). To suppress aspartimide formation, Fmoc-Asp(OMpe)-OH was used for the Asp_75_, Asp_104_, and Asp_108_ residues instead of standard Fmoc-Asp(OtBu)-OH. The resin was washed with DMF and DCM, dried in vacuo and treated with a cleavage cocktail (95% TFA, 2.5% TIPS, 2.5% H_2_O) for global deprotection and release of the peptide. The mixture was stirred at room temperature for 2 h. Then, the solution was filtered to remove the resin, and ten volumes of cold diethyl ether were added to the filtrate to precipitate the peptide. This suspension was vortexed and centrifuged at 10,000 rpm at 4 °C for 10 min. The supernatant was removed by decantation. Obtained pellet was washed twice with diethyl ether. The crude peptide was dissolved in water/acetonitrile containing 0.1% TFA and purified by semi-preparative RP-HPLC. After lyophilization, peptide **5** was obtained as a white solid (55.2 mg, 6.9 μmol, 14%). HPLC profile and mass data after purification are shown in Figure S7.

**1.3.3 Preparation of model peptide**

**Peptide synthesis of TKULYRV-NH_2_ (11)**

TKULYRV-NH2 (**11**) was prepared with Rink amide Resin (Watanabe chemical, 0.50 mmol/g, 5 μmol scale). This peptide was elongated by MultiPep RS (Intavis). Fmoc-Sec(PMB)-OH was used for the Sec residue. The resin was washed with DMF and DCM, dried in vacuo and treated with a cleavage cocktail (90% TFA, 2.5% TIPS, 2.5% H_2_O, 5.0% DMB including 2 equiv. of 2,2'-Dithiobis(5-nitropyridine) (DTNP) and 5 equiv. of MeONH_2_·HCl) for global deprotection, release of the peptide, and the deprotection of PMB [66]. The mixture was stirred at room temperature for 2 h. Then, the solution was filtered to remove the resin, and ten volumes of cold diethyl ether were added to the filtrate to precipitate the peptide. This suspension was vortexed and centrifuged at 10,000 rpm at 4 °C for 10 min. The supernatant was removed by decantation. Obtained pellet was washed five times with diethyl ether. The crude peptide was dissolved in water/acetonitrile containing 0.1% TFA and purified by semi-preparative RP-HPLC. After lyophilization, peptide **11** was obtained as a white solid (1.67 mg, 1.57 μmol, 31%). HPLC profile and mass data after purification are shown in Figure S14.

**1.3.4 Preparation of biotinylated peptides**

**Peptide synthesis of U-PAF15(33–37)-(spacer)-K(Biotin)-NH_2_ (13)**

U-PAF15(33–37)-(spacer)-K(Biotin)-NH_2_ (**13**) was prepared with Rink amide Resin (Watanabe chemical, 0.50 mmol/g, 35 μmol scale). This peptide was elongated by Initiator+ Alstra (Biotage). As a spacer, Fmoc-NH-PEG2-DGA-OH (Watanabe Chemical) was introduced. Fmoc-Sec(PMB)-OH was used for the Sec residue. The resin was washed with DMF and DCM, dried in vacuo and treated with a cleavage cocktail (90% TFA, 2.5% TIPS, 2.5% H_2_O, 5.0% DMB including 5 equiv. of 2,2'-Dithiobis(5-nitropyridine) (DTNP) and 5 equiv. of MeONH_2_·HCl) for global deprotection, release of the peptide, and the deprotection of PMB [66]. The mixture was stirred at room temperature for 2 h. For the last 5 min of the cleavage, dimethyl sulfide (10% v/v) and tetrabutylammonium iodide (TBAI, 1% w/v) were added to the TFA cocktail to reduce the oxidized biotin moiety. Then, the solution was filtered to remove the resin, and ten volumes of cold diethyl ether were added to the filtrate to precipitate the peptide. This suspension was vortexed and centrifuged at 10,000 rpm at 4 °C for 10 min. The supernatant was removed by decantation. Obtained pellet was washed five times with diethyl ether. The crude peptide was dissolved in water/acetonitrile containing 0.1% TFA and purified by semi-preparative RP-HPLC. After lyophilization, peptide **13** was obtained as a white solid (28.0 mg, 19.4 μmol, 55%). HPLC profile and mass data after purification are shown in Figure S16.

**Peptide synthesis of PAF15(2–37)-(spacer)-K(Biotin)-NH_2_ (15)**

PAF15(2–37)-(spacer)-K(Biotin)-NH_2_ (**15**) was prepared with Rink amide-PEG Resin (Watanabe chemical, 0.22 mmol/g, 70 μmol scale). This peptide was elongated by Initiator+ Alstra (Biotage). As a spacer, Fmoc-NH-PEG2-DGA-OH (Watanabe Chemical) was introduced. The resin was washed with DMF and DCM, dried in vacuo and treated with a cleavage cocktail (95% TFA, 2.5% TIPS, 2.5% H_2_O, 5.0% DMB) for global deprotection and release of the peptide. The mixture was stirred at room temperature for 2 h. For the last 5 min of the cleavage, dimethyl sulfide (10% vol/vol) and tetrabutylammonium iodide (TBAI, 1% wt/vol) were added to the TFA cocktail to reduce the oxidized biotin moiety. Then, the solution was filtered to remove the resin, and ten volumes of cold diethyl ether were added to the filtrate to precipitate the peptide. This suspension was vortexed and centrifuged at 10,000 rpm at 4 °C for 10 min. The supernatant was removed by decantation. Obtained pellet was washed five times with diethyl ether. The crude peptide was dissolved in water/acetonitrile containing 0.1% TFA and purified by semi-preparative RP-HPLC. After lyophilization, peptide **15** was obtained as a white solid (48.4 mg, 9.12 μmol, 13%). HPLC profile and mass data after purification are shown in Figure S17.

1.4 **Chemical synthesis of PAF15Ub2**

**1.4.1. One-pot ligation of 1, 2 and 3 (7)**

Peptide **2** (1.11 mg, 161 nmol, 1.1 equiv.) and peptide **3** (0.90 mg, 150 nmol, 1.0 equiv.) were dissolved in 50 μL of NCL buffer (8 M Gn·HCl, 100 mM MPAA, 20 mM TCEP·HCl, 0.2 M phosphate (pH 7.0); peptide ~2 mM). The mixture was stirred at 37 °C for 1 h under an argon atmosphere (1st NCL, Figure 3A). Thz deprotection was performed according to a previous report [44]. 2.1 mg of 2-amino-5-methoxy-*N',N'*-dimethylbenzohydrazide (AMDBH) was added to the reaction mixture, and the pH of the reaction mixture was adjusted to ~4 by adding 6 M HCl to convert N-terminal Thz into cysteine. After stirring for 17 h at 37 °C under an argon atmosphere, an additional 1.1 mg of AMDBH was added (final 300 mM). The mixture was stirred for another 40 h (Thz deprotection, Figure 3A). Then, peptide **1** (1.73 mg, 285 nmol, 1.9 equiv.) and 4 µL of 500 mM TCEP·HCl (pH 7.0) was added to the reaction mixture, and the pH of the solution was adjusted to ~6.5 with 1 M NaOH. The reaction solution was stirred at 37 °C for 3 h under an argon atmosphere (2nd NCL, Figure 3A). For analysis of each reaction, an aliquot (0.6 μL) of each reaction mixture was treated with 0.6 μL of 500 mM TCEP·HCl (pH 7.0) and 44 µL of NCL buffer (pH 3.0), then injected into the analytical RP-HPLC system. Finally, 50 μL of 500 mM TCEP·HCl (pH 7.0) was added to the reaction mixture and the solution was stirred at room temperature for 10 min. The peptide solution was diluted with NCL buffer (pH 3.0) and a mixture of water/acetonitrile containing 0.1% TFA, purified by RP-HPLC and identified by ESI–MS. After lyophilization, peptide **7** was obtained as a white solid (1.29 mg, 53.9 nmol, 38% isolated yield). HPLC profile and mass data after purification are shown in Figure S9.

**1.4.2. Desulfurization (8)**

The desulfurization reaction was performed according to a previous report [34]. Peptide **7** (1.29 mg, 53.9 nmol) was dissolved in 220 μL of the buffer containing TCEP·HCl, glutathione, Gn·HCl, and sodium phosphate. 1.42 mg of VA-044 was added to the reaction solution (final concentrations: 0.25 mM peptide, 300 mM TCEP·HCl, 80 mM glutathione, 20 mM VA-044, 8M Gn·HCl, 0.2 M sodium phosphate, at pH 7.0), and the reaction mixture was stirred at 37 °C for 20 h under an argon atmosphere (Figure 3B). For analysis of each reaction, an aliquot (3.5 μL) of each reaction mixture was treated with 3.5 μL of 500 mM TCEP·HCl (pH 7.0) and 40 µL of NCL buffer (pH 3.0), then injected into the analytical HPLC system. Then, the peptide solution was diluted with water/acetonitrile containing 0.1% TFA, purified by preparative RP-HPLC and identified by ESI–MS. After lyophilization, peptide **8** was obtained as a white solid (26.2 nmol, 49% isolated yield). HPLC analysis and mass data after purification are shown in Figure 3B and S10.

**1.4.3. Ligation of 4 and 5 and Sez deprotection (9)**

Peptide **4** (0.64 mg, 225 nmol, 1.5 equiv.) and peptide **5** (1.20 mg, 150 nmol, 1.0 equiv.) were dissolved in 42 μL of NCL buffer (8 M Gn·HCl, 140 mM MPAA, 30 mM TCEP·HCl, 140 mM sodium ascorbate, 0.2 M phosphate (pH 7.0); 5.4 mM peptide **4**, 3.6 mM peptide **5**). The mixture was stirred at 37 °C for 7 h under an argon atmosphere, then an additional 0.8 equiv. of peptide **4** and 1.68 µL of 500 mM TCEP·HCl (pH 7.0) were added. The mixture was stirred for another 15 h (NCL, Figure 3C). 0.75 mg of MeONH_2_ was added to the reaction mixture, and the pH of the reaction mixture was adjusted to ~3 by adding 6 M HCl. The mixture was stirred for 7 h at 37 °C under an argon atmosphere to convert N-terminal Sez into selenocysteine (Sez deprotection). For analysis of each reaction, an aliquot (0.7 μL) of each reaction mixture was treated with 0.7 µL of 1 M sodium ascorbate in 0.2 M phosphate buffer (pH7.0) and 1.5 μL of 100 mM TCEP·HCl (pH 7.0), and then injected into the analytical RP-HPLC system. Finally, 40 μL of 1 M sodium ascorbate in 0.2 M phosphate buffer (pH7.0) and 80 µL of 100 mM TCEP·HCl (pH 7.0) were added to the reaction mixture and the solution was stirred at room temperature for 10 min. The peptide solution was diluted with a mixture of water/acetonitrile containing 0.1% TFA, purified by RP-HPLC and identified by ESI–MS. After lyophilization, peptide **9** was obtained as a white solid (1.04 mg, 97.9 nmol, 65% isolated yield). HPLC profile and mass data after purification are shown in Figure 3C.

**1.4.4. Ligation of 8 and 9 and deselenization (10)**

Peptide **8** (0.58 mg, 24.0 nmol, 1.0 equiv.) was dissolved in 15 μL of NCL buffer (8 M Gn·HCl, 100 mM MPAA, 0.2 M phosphate (pH 3.0); peptide 1.6 mM). 150 mM acetylacetone in H_2_O (5.0 equiv.) was added to the reaction mixture and the mixture was stirred at 37 °C for 2 h (MPAA thioesterification, Figure 3E). Peptide **9** (0.32 mg, 30.0 nmol, 1.25 equiv.) was added to the mixture and the pH of the mixture was adjusted to ~6.5 by adding 1 M NaOH. The mixture was stirred for 8.5 h at 37 °C under an argon atmosphere (NCL, Figure 3E). 2.6 mg (300 equiv.) of TCEP·HCl was added to the reaction mixture and the pH of the reaction mixture was adjusted to ~5 by adding 6 M HCl. The mixture was stirred for 36 h at 37 °C under an argon atmosphere to convert the selenocysteine residue into alanine (deselenization, Figure 3E). For analysis of each reaction, an aliquot (0.7 μL) of each reaction mixture was treated with 0.7 µL of 1 M sodium ascorbate in 0.2 M phosphate buffer (pH7.0) and 1.5 μL of 100 mM TCEP·HCl (pH 7.0), and then injected into the analytical RP-HPLC system. Finally, 15 µL of 100 mM TCEP·HCl (pH 7.0) was added to the reaction mixture and the solution was stirred at room temperature for 10 min. The peptide solution was diluted with a mixture of water/acetonitrile containing 0.1% TFA, purified by RP-HPLC and identified by ESI–MS. After lyophilization, peptide **10** was obtained as a white solid (3.14 nmol, 13% isolated yield). HPLC profile and mass data after purification are shown in Figure 3E.

**1.5 Deselenization of model peptide 11**

Peptide **11** (40 nmol, 1.0 equiv.) and MPAA (0, 1, or 100 equiv.) were dissolved in 20 μL of degassed buffer containing Gn·HCl and sodium phosphate (pH 7.0) and the pH of the mixture was adjusted to 5.0. TCEP·HCl (20 µL, 600 mM aqueous solution, pH 5.0) was added to the mixture to initiate the deselenization reaction (final concentrations: 1.0 mM peptide, 300 mM TCEP·HCl, 4 M Gn·HCl, 0.1 M sodium phosphate, pH 5.0). The mixture was stirred at 50 °C for 30 min under an argon atmosphere. For analysis of each reaction, an aliquot (2 μL) of each reaction mixture was quenched with 2 µL of 1 M sodium ascorbate in 0.2 M sodium phosphate buffer (pH 7.0) and 96 μL of H_2_O containing 0.1% TFA, and then injected into the analytical RP-HPLC system.

**1.6 Electrophoretic mobility shift assay (EMSA)**

Recombinant RFTS domain of human DNMT1, residues 351-600, was expressed in *E. coli* and purified as previously described [9]. The RFTS domain and synthetic PAF15Ub2 (peptide **8** or **10**) were incubated for 30 min at 4°C in a binding buffer [final concentrations: 1 µM RFTS domain, 0, 0.5, 1 or 2 µM PAF15Ub2 peptide (**8** or **10**), 20 mM Tris-HCl (pH 7.5), 150 mM NaCl, 0.05% NP-40, 1 mM DTT and 10% (w/v) glycerol]. To analyze the interactions, electrophoresis was performed using a Tris-borate buffer [25 mM Tris containing 12.5 mM boric acid (pH 8.8)] at a constant current of 10 mA for 40 min on ice using an 8% polyacrylamide gel. Proteins were detected by staining with Oriole^TM^ and analyzed using ChemiDoc Touch MP system (Bio-Rad).

**1.7 Preparation of biotinylated N-terminal segment of PAF15Ub2 (14)**

Peptide **8** (10 nmol, 1.0 equiv.) was dissolved in 10 μL of NCL buffer (8 M Gn·HCl, 100 mM MPAA, 0.2 M phosphate (pH 3.0); peptide 1.0 mM). 25 mM acetylacetone in H_2_O (2.5 equiv.) was added to the reaction mixture, and the mixture was stirred at 37 °C for 1.5 h (MPAA thioesterification, Figure S18B). Biotinylated peptide **13** (0.10 mg, 70 nmol, 7.0 equiv.) was added to the mixture and the pH of the mixture was adjusted to ~7 by adding 1 M NaOH. The mixture was stirred for 2 h at 37 °C under an argon atmosphere (NCL, Figure S18B). 0.85 mg (300 equiv.) of TCEP·HCl was added to the reaction mixture and the pH of the reaction mixture was adjusted to ~7 by adding 6 M HCl. The mixture was stirred for 3 h at 37 °C under an argon atmosphere to convert the selenocysteine residue into alanine (deselenization, Figure S18B). For analysis of each reaction, an aliquot (0.5 μL) of each reaction mixture was treated with 0.5 µL of 1 M sodium ascorbate in 0.2 M phosphate buffer (pH7.0) and 1.0 μL of 100 mM TCEP·HCl (pH 7.0), and then injected into the LC–MS system. The peptide solution was diluted with a mixture of water/acetonitrile containing 0.1% TFA, purified by RP-HPLC and identified by ESI–MS. After lyophilization, peptide **14** was obtained as a white solid (6.80 nmol, 68% isolated yield). HPLC profile and mass data after purification are shown in Figure S18B and S19.

**1.8 Affinity measurement of PAF15Ub2 against hRFTS domain by BLI**

Affinity measurements were performed on biotinylated peptide **14** or **15** immobilized on a streptavidin biosensor (ForteBio) using the Octet system (ForteBio) according to the manufacturer’s instructions. The assay buffer consisted of 50 mM HEPES (pH 7.5), 300 mM NaCl, 0.05% (vol/vol) Tween 20, and 0.1% (wt/vol) PEG. The hRFTS domain was diluted with water and buffer-exchanged into the assay buffer using Zeba™ Spin Desalting Columns. Solutions of peptides **14** and **15** were stored at −80 °C and diluted with the assay buffer before use. The binding assay was performed at 30 °C in the assay buffer, with the following steps: equilibration for 60 s, association for 600 s, and dissociation for 600 s.

**1.9 *in vitro* DNA methylation assay**

Recombinant human DNMT1, residues 351-1616, was expressed in Sf-9 cells using the baculovirus system and purified according to a previous report [10]. Recombinant PAF15 with a C-terminal FLAG tag, and ubiquitin were expressed in *E. coli* BL21 (DE3) and purified according to the previous reports [48, 9]. A 42 bp DNA duplex containing three hemimethylation sites (upper: 5′-GGACATC(mC)GTGAGATCGGAGGC(mC)GCCTGCTGCAATC(mC)GGTAG, lower: 5′-CTACCGGATTGCAGCAGGCGGCCTCCGATCTCACGGATGTCC, 0−0.8 µM) was methylated with 15 nM DNMT1 by the addition of a 10-fold molar excess of chemically synthesized PAF15Ub2 including 20 µM S-adenosyl-L-methionine (SAM) in reaction buffer [20 mM Tris-HCl (pH 8.0), 50 mM NaCl, 1 mM EDTA, 3 mM MgCl_2_, 0.1 mg/mL BSA, and 20% Glycerol] at 37 °C for 1 h. DNA methylation activity was measured using MTase-Glo^TM^ (Promega) and monitored using GloMax® Navigator Microplate Luminometer (Promega).

**1.10 Cryo-EM analysis**

**1.10.1 Preparation of ternary complex for cryo-EM single particle analysis**

For preparation of DNMT1:PAF15Ub2:DNA^mCG/fCG^ ternary complex, 20 μM synthetic PAF15Ub2 segment 8 was mixed with 15 μM DNMT1, residues 351-1616 in the conjugation buffer [50 mM Tris-HCl (pH 7.5), 20% glycerol, 5 mM DTT and 50 mM NaCl]. After 30 min incubation on ice, a 12 bp hemimethylated DNA analog (upper: 5’-ACTTA(mC)GGAAGG, lower: 5’-CCTTC(fC)GTAAGT: DNA^mCG/fCG^) was added to the protein solution. The conjugation reaction was initiated by the addition of 500 μM SAM at 37 °C overnight. The product was purified by size-exclusion chromatography (SEC) using Superdex^TM^ 200 Increase 10/300 GL equilibrated with cryo-EM buffer (20 mM Tris-HCl [pH 7.5], 100 mM NaCl and 5 mM DTT). The SEC chromatogram and gel analyses of the purified complex are shown in Figure S20.

**1.10.2 Data collection**

A 3 µL aliquot of the protein solution was applied onto the glow-discharged holey carbon grids (Quantifoil Cu R1.2/1.3, 300 mesh). The grids were plunge-frozen in liquid ethane using a Vitrobot Mark IV (Thermo Fisher Scientific). Parameters for plunge-freezing were set as follows: blotting time, 3 s; waiting time, 3 s; blotting force, 5; humidity, 100%; and chamber temperature, 4 °C. Data for DNMT1:PAF15Ub2:DNA^mCG/fCG^ ternary complex were collected at RIKEN BDR on a 300 kV Krios G4 (Thermo Fisher Scientific) with a K3 direct electron detector (Gatan) with BioQuantum energy filter (slit width 15 eV). A total of 4,656 movies were recorded at a nominal magnification of ×105,000 with a pixel size of 0.83 Å, in a total exposure of 60.848 e^−^/Å^2^ per 48 frames with an exposure time of 2.2 s. The data were automatically acquired by the image shift method of the EPU software (Version 3.2.0.4776REL, Thermo Fisher Scientific), with a defocus range of −0.6 to −2.2 μm.

**1.10.3 Data processing**

All data were processed using cryoSPARC v4.0.3 [67] for PDB deposition. The movie stacks were motion corrected by Patch Motion Correction. The defocus values were estimated from Contrast transfer function (CTF) by Patch CTF Estimation. Micrographs with CTF resolutions exceeding 8 Å were discarded using Curate Exposures, and 4,516 micrographs were selected. A total of 3,852,575 particles were automatically picked using Blob Picker and the junk particles were removed using Inspect Particle Picks. Particles (2,385,837) were extracted from micrographs with a box size of 256 px, which were reduced to 64 px (3.31 Å/px) and these particles were subjected to a single round of 2D Classifications. Particles were further curated by Heterogeneous Refinement using the three maps derived from cryoSPARC *Ab-Initio* Reconstruction as the templates. The selected suitable class containing 445,931 particles was extracted with a box size of 256 px, which were reduced to 128 px (1.66 Å/px). 356,895 particles were selected by subsequent two rounds of Heterogeneous Refinement. These particles were re-extracted with the 360 px box size and downsampled to 250 px (1.20 Å/px). 3D reconstruction was performed using Non-uniform Refinement, and a 3.25 Å resolution map was finally obtained according to the gold-standard Fourier shell correlation (FSC) = 0.143 criterion. Details of the data processing are shown in Figure S21.

**1.10.4 Model building and validation**

The previously reported structure of the DNMT1:H3Ub2:DNA^mCG/fCG^ ternary complex (PDB ID: 7XI9) was used as the initial model. The model was roughly fitted to the cryo-EM map by UCSF ChimeraX version 1.5 [68] and then refined using Real-Space Refine in PHENIX version 1.20.1-4487 [69]. The model was modified using Coot version 0.9.4.1 [70] and refined using Real-Space Refine in PHENIX for further refinement. Refinement statistics are shown in Table S1.

**Table S1. Cryo-EM data collection and refinement statistics for DNMT1:PAF15Ub2:DNA^mCG/fCG^**

|  | **DNMT1:PAF15Ub2:DNA^mCG/fCG^** |
| --- | --- |
| EMDB number | EMD-38573 |
| PDB number | 8XQC |
| **Data-collection parameters** | |
| Magnification | 105,000× |
| Voltage (kV) | 300 |
| Total electron exposure/frame (e^-^/Å^2^) | 60.848 |
| Defocus range (μm) | -0.6 to -2.2 |
| Pixel size (Å) | 0.8285 |
| Symmetry imposed | *C*1 |
| Number of images | 4,656 |
| Initial particle images (no.) | 2,385,837 |
| Final particle images (no.) | 356,895 |
| Map Resolution (Å)  FSC threshold | 3.25  0.143 |
| **Refinement** | |
| Initial models used | 7XI9 |
| Model resolution (Å)  FSC threshold | 3.40  0.5 |
| Map sharpening *B* factor (Å^2^) | -100 |
| Model composition  Non-hydrogen atoms  Protein residues  Nucleotide  Ion (zinc) | 7,233  843  24  2 |
| *B* factors (Å^2^)  Protein (min/max/mean)  Nucleotide (min/max/mean)  Ligand (min/max/mean) | 9.97/149.12/73.43  72.38/85.30/78.27  58.16/163.76/109.85 |
| CC (volume/mask) | 0.80/0.81 |
| R.m.s. deviations  Bond lengths (Å)  Bond angles (°) | 0.003  0.562 |
| Validation  MolProbity score  CaBLAM outliers  Clashscore  Poor rotamers (%)  Rama-Z score | 1.68  1.12  7.09  0.41  -0.11 |
| Ramachandran plot  Favored (%)  Allowed (%)  Disallowed (%) | 95.87  4.13  0.00 |

2. **Supporting Figures**

**Figure S1.** *in vitro* ubiquitination of PAF15-FLAG by UHRF1. **(A)** Amino-acid sequence of PAF15_2-71_-FLAG. Targeted Lys residues are shown in green, and the other Lys residues are shown in red. **(B)** SDS-PAGE analysis of *in vitro* ubiquitination of PAF15_2-71_-FLAG by UHRF1.

**Figure S2**. 1st-generation synthesis of di-monoubiquitinated PAF15 (**X11**). **(A)** Synthetic scheme of di-monoubiquitinated PAF15 (**X11**). **(B)** RP-HPLC analysis of the final NCL. Fraction **a** and **b** were collected separately. Column: Jupiter 5C4 300Å Column (4.6 ID × 250) (Phenomenex). Gradient: acetonitrile 25–45% for 30 min. (C) SDS-PAGE analysis of fraction **a** and **b**.

**Figure S3**. RP-HPLC analysis and ESI-MS spectrum of peptide **1** for the chemical synthesis of di-monoubiquitinated PAF15. **(A)** RP-HPLC profile of purified peptide **1.** Column: COSMOSIL Protein-R Packed Column (4.6 ID × 250) (nacalai). Gradient: acetonitrile 15–45% for 30 min. **(B)** ESI-MS spectrum, Sequence: H−MQIFVKTLTGKTITLEVEPSDTIENVKAKIQDKEGIPPDQQRLIF−MPAA thioester, MS(ESI) *m/z*: [M+7H]^7+^ Calcd 753.0; Found 752.9 [M+6H]^6+^ Calcd 878.4; Found 878.2, [M+5H]^5+^ Calcd 1053.8; Found 1053.6, [M+4H]^4+^ Calcd 1317.0; Found 1316.8. The deconvoluted MS(ESI): Calcd for C_237_H_386_N_58_O_72_S_2_ 5264.1; Found 5263.4. Deconvoluted masses were obtained using the deconvolution function in Agilent ChemStation.

**Figure S4**. RP-HPLC analysis and ESI-MS spectrum of peptide **2** for the chemical synthesis of di-monoubiquitinated PAF15. **(A)** RP-HPLC profile of purified peptide **2.** Column: COSMOSIL Protein-R Packed Column (4.6 ID × 250) (nacalai). Gradient: acetonitrile 15–45% for 30 min. **(B)** ESI-MS spectrum, Sequence: H−VRTKADSVPGTYRK(Thz−GKQLEDGRTLSDYNIQKESTLHLVLRLRGG)VVA−MPAA thioester, MS(ESI) *m/z*: [M+10H]^10+^ Calcd 550.2; Found 550.1, [M+9H]^9+^ Calcd 611.3; Found 611.2, [M+8H]^8+^ Calcd 687.5; Found 687.4, [M+7H]^7+^ Calcd 785.6; Found 785.5, [M+6H]^6+^ Calcd 916.4; Found 916.2, [M+5H]^5+^ Calcd 1099.5; Found 1099.3. The deconvoluted MS(ESI): Calcd for C_239_H_394_N_72_O_72_S_2_ 5492.3; Found 5491.5. Deconvoluted masses were obtained using the deconvolution function in Agilent ChemStation.

**Figure S5**. RP-HPLC analysis and ESI-MS spectrum of peptide **3** for the chemical synthesis of di-monoubiquitinated PAF15. **(A)** RP-HPLC profile of purified peptide **3.** Column: COSMOSIL Protein-R Packed Column (4.6 ID × 250) (nacalai). Gradient: acetonitrile 15–45% for 30 min. **(B)** ESI-MS spectrum, Sequence: H−CRAPRK(Thz−GKQLEDGRTLSDYNIQKESTLHLVLRLRGG)VLGSSTS−NHNH_2_, MS(ESI) *m/z*: [M+9H]^9+^ Calcd 542.2; Found 542.0, [M+8H]^8+^ Calcd 609.8; Found 609.7, [M+7H]^7+^ Calcd 696.8; Found 696.7, [M+6H]^6+^ Calcd 812.8; Found 812.6, [M+5H]^5+^ Calcd 975.1; Found 974.9, [M+4H]^4+^ Calcd 1218.6; Found 1218.4, The deconvoluted MS(ESI): Calcd for C_205_H_351_N_69_O_64_S_2_ 4870.5; Found 4869.7. Deconvoluted masses were obtained using the deconvolution function in Agilent ChemStation.

**Figure S6**. RP-HPLC analysis and ESI-MS spectrum of peptide **4** for the chemical synthesis of di-monoubiquitinated PAF15. **(A)** RP-HPLC profile of purified peptide **4.** Column: COSMOSIL 5C18-AR-Ⅱ Packed Column (4.6 ID × 250) (nacalai). Gradient: acetonitrile 5–30% for 30 min. **(B)** ESI-MS spectrum, Sequence: Sez−TNSTSVSSRKAENKYAGGNPV−MPAA thioester, MS(ESI) *m/z*: [M+4H]^4+^ Calcd 620.9; Found 620.9, [M+3H]^3+^ Calcd 827.5; Found 827.5, [M+2H]^2+^ Calcd 1240.8; Found 1240.6. The deconvoluted MS(ESI): Calcd for C_101_H_158_N_30_O_36_S_1_Se_1_ 2479.5; Found 2479.4. Deconvoluted masses were obtained using the deconvolution function in Agilent ChemStation.

**Figure S7**. RP-HPLC analysis and ESI-MS spectrum of peptide **5** for the chemical synthesis of di-monoubiquitinated PAF15. **(A)** RP-HPLC profile of purified peptide **5.** Column: COSMOSIL Protein-R Packed Column (4.6 ID × 250) (nacalai). Gradient: acetonitrile 15–45% for 30 min. **(B)** ESI-MS spectrum, Sequence: H−CVRPTPKWQKGIGEFFRLSPKDSEKENQIPEEAGSSGLGKAKRKACPLQPDHTNDEKE−OH, MS(ESI) *m/z*: [M+11H]^11+^ Calcd 591.4; Found 591.3, [M+10H]^10+^ Calcd 650.4; Found 650.3, [M+9H]^9+^ Calcd 722.6; Found 722.5, [M+8H]^8+^ Calcd 812.8; Found 812.6, [M+7H]^7+^ Calcd 928.8; Found 928.6, [M+6H]^6+^ Calcd 1083.4; Found 1083.2. The deconvoluted MS(ESI): Calcd for C_281_H_449_N_83_O_90_S_2_ 6494.2; Found 6493.1. Deconvoluted masses were obtained using the deconvolution function in Agilent ChemStation.

**Figure S8**. ESI-MS spectrum of peptide **6**. Sequence: H−VRTKADSVPGTYRK(CGKQLEDGRTLSDYNIQKESTLHLVLRLRGG)VVACRAPRK(CGKQLEDGRTLSDYNIQKESTLHLVLRLRGG)VLGSSTS−NHNH_2_, MS(ESI) *m/z*: [M+14H]^14+^ Calcd 727.5; Found 727.4, [M+13H]^13+^ Calcd 783.4; Found 783.3, [M+12H]^12+^ Calcd 848.6; Found 848.5, [M+11H]^11+^ Calcd 925.6; Found 925.5, [M+10H]^10+^ Calcd 1018.1; Found 1017.9, [M+9H]^9+^ Calcd 1131.1; Found 1130.9. The deconvoluted MS(ESI): Calcd for C_434_H_737_N_141_O_134_S_3_ 10170.6; Found 10169.9. Deconvoluted masses were obtained using the deconvolution function in Agilent ChemStation.

**Figure S9**. RP-HPLC analysis and ESI-MS spectrum of peptide **7**. **(A)** RP-HPLC profile of purified peptide **7.** Column: Jupiter 5C4 300Å Column (4.6 mm i.d. × 250 mm) (Phenomenex). Gradient: acetonitrile 15–45% for 30 min. **(B)** ESI-MS spectrum, Sequence: H−VRTKADSVPGTYRK(MQIFVKTLTGKTITLEVEPSDTIENVKAKIQDKEGIPPDQQRLIFCGKQLEDGRTLSDYNIQKESTLHLVLRLRGG)VVACRAPRK(MQIFVKTLTGKTITLEVEPSDTIENVKAKIQDKEGIPPDQQRLIFCGKQLEDGRTLSDYNIQKESTLHLVLRLRGG)VLGSSTS−NHNH_2_, MS(ESI) *m/z*: [M+26H]^26+^ Calcd 784.2; Found 784.1, [M+25H]^25+^ Calcd 815.5; Found 815.4, [M+24H]^24+^ Calcd 849.4; Found 849.3, [M+23H]^23+^ Calcd 886.3; Found 886.2, [M+22H]^22+^ Calcd 926.6; Found 926.4, [M+21H]^21+^ Calcd 970.6; Found 970.5. The deconvoluted MS(ESI): Calcd for C_892_H_1493_N_257_O_274_S_5_ 20362.3; Found 20360.9. Deconvoluted masses were obtained using the deconvolution function in Agilent ChemStation.

**Figure S10**. ESI-MS spectrum of peptide **8**. Sequence: H−VRTKADSVPGTYRK(AGKQLEDGRTLSDYNIQKESTLHLVLRLRGG)VVAARAPRK(AGKQLEDGRTLSDYNIQKESTLHLVLRLRGG)VLGSSTS−NHNH_2_, MS(ESI) *m/z*: [M+21H]^21+^ Calcd 966.1; Found 965.9, [M+20H]^20+^ Calcd 1014.3; Found 1014.2, [M+19H]^19+^ Calcd 1067.6; Found 1067.5, [M+18H]^18+^ Calcd 1126.9; Found 1126.7, [M+17H]^17+^ Calcd 1193.1; Found 1193.0, [M+16H]^16+^ Calcd 1267.6; Found 1267.5. The deconvoluted MS(ESI): Calcd for C_892_H_1493_N_257_O_274_S_2_ 20266.1; Found 20264.1. Deconvoluted masses were obtained using UniDec.

**Figure S11**. ESI-MS spectrum of peptide **9**. Sequence: H− UTNSTSVSSRKAENKYAGGNPVCVRPTPKWQKGIGEFFRLSPKDSEKENQIPEEAGSSGLGKAKRKACPLQPDHTNDEKE −OH, MS(ESI) *m/z*: [M+12H]^12+^ Calcd 733.8; Found 733.6, [M+11H]^11+^ Calcd 800.4; Found 800.2, [M+10H]^10+^ Calcd 880.4; Found 880.1, [M+9H]^9+^ Calcd 978.1; Found 977.8, [M+8H]^8+^ Calcd 1100.2; Found 1099.9, [M+7H]^7+^ Calcd 1257.2; Found 1256.8, The deconvoluted MS(ESI): Calcd for C_373_H_599_N_113_O_124_S_2_Se_1_ 8793.5; Found 8790.9. Deconvoluted masses were obtained using UniDec.

**Figure S12**. ESI-MS spectrum of peptide **8’**. Sequence: H−VRTKADSVPGTYRK(AGKQLEDGRTLSDYNIQKESTLHLVLRLRGG)VVAARAPRK(AGKQLEDGRTLSDYNIQKESTLHLVLRLRGG)VLGSSTS−MPAA, MS(ESI) *m/z*: [M+21H]^21+^ Calcd 972.5; Found 972.4, [M+20H]^20+^ Calcd 1021.1; Found 1021.1, [M+19H]^19+^ Calcd 1074.8; Found 1074.7, [M+18H]^18+^ Calcd 1134.5; Found 1134.3, [M+17H]^17+^ Calcd 1201.1; Found 1201.0, [M+16H]^16+^ Calcd 1276.1; Found 1276.0. The deconvoluted MS(ESI): Calcd for C_900_H_1497_N_255_O_276_S_3_ 20402.3; Found 20400.6. Deconvoluted masses were obtained using the deconvolution function in Agilent ChemStation.

**Figure S13**. ESI-MS spectrum of peptide **10**. Sequence: H−VRTKADSVPGTYRK(AGKQLEDGRTLSDYNIQKESTLHLVLRLRGG)VVAARAPRK(AGKQLEDGRTLSDYNIQKESTLHLVLRLRGG)VLGSSTSATNSTSVSSRKAENKYAGGNPVCVRPTPKWQKGIGEFFRLSPKDSEKENQIPEEAGSSGLGKAKRKACPLQPDHTNDEKE−OH, MS(ESI) *m/z*: [M+30H]^30+^ Calcd 965.9; Found 965.6, [M+29H]^29+^ Calcd 999.2; Found 999.1, [M+28H]^28+^ Calcd 1034.9; Found 1034.7. [M+27H]^27+^ Calcd 1073.2; Found 1073.0. The deconvoluted MS(ESI): Calcd for C_1265_H_2088_N_368_O_398_S_4_ 28948.3; Found 28945.2. Deconvoluted masses were obtained using UniDec.

**Figure S14**. RP-HPLC analysis and ESI-MS spectrum of peptide **11**. **(A)** RP-HPLC profile of purified peptide **11.** Column: 5C18 AR-II column (4.6 mm i.d. × 250 mm) (Nacalai). Gradient: acetonitrile 5–35% for 30 min. **(B)** MALDI-TOF MS spectrum of peptide **11**. Sequence: H−TKULYRV−NH_2_, MS(MALDI-TOF) *m/z*: [M+H]^+^ Calcd for C_39_H_68_N_12_O_9_Se 929.4; Found 929.5.

**Figure S15**. Deselenization test of model peptide **11**. **(A)** Time course RP-HPLC analysis of deselenization reaction of **11** in the presence of 0, 1, or 100 mM MPAA. Column: 5C18 AR-II column (4.6 mm i.d. × 250 mm) (Nacalai). Gradient: acetonitrile 5–35% for 30 min. **(B)** MALDI-TOF MS spectrum of **11**. Sequence: H−TKULYRV−NH_2_, MS(MALDI-TOF) *m/z*: [M+H]^+^ Calcd for C_39_H_68_N_12_O_9_Se 929.4; Found 929.2. **(C)** MALDI-TOF MS spectrum of **12**. Sequence: H−TKALYRV−NH_2_, MS(MALDI-TOF) *m/z*: [M+H]^+^ Calcd for C_39_H_68_N_12_O_9_ 849.5; Found 849.3.

**Figure S16** RP-HPLC analysis and ESI-MS spectrum of peptide **13**. The peptide was observed as the diselenide-linked dimer. **(A)** RP-HPLC profile of purified peptide **13.** Column: ZORBAX 300SB-C8 3.5 µm (3.0 mm × 100 mm) (Agilent). Gradient: acetonitrile 1–30% over 20 min. **(B)** ESI-MS spectrum, Sequence: UTNSTS−(spacer)−Lys(biotin)−NH_2_, MS(ESI) *m/z*: [M_2_+4H]^4+^ Calcd 665.7; Found 665.6, [M_2_+3H]^3+^ Calcd 887.2; Found 887.3, [M_2_+2H]^2+^ Calcd 1330.4; Found 1330.8. The deconvoluted MS(ESI): Calcd for C_102_H_178_N_28_O_40_S_2_Se_2_ (M_2_) 2658.7; Found 2658.4. Deconvoluted masses were obtained using the deconvolution function in Agilent ChemStation.

**Figure S17**. RP-HPLC analysis and ESI-MS spectrum of peptide **15**. **(A)** RP-HPLC profile of purified peptide **15.** Column: 5C18 AR-II column (4.6 mm i.d. × 250 mm) (Nacalai). Gradient: acetonitrile 5–35% over 30 min. **(B)** ESI-MS spectrum, Sequence: H− VRTKADSVPGTYRKVVAARAPRKVLGSSTSATNSTS−(spacer)−Lys(biotin)−NH_2_, MS(ESI) *m/z*: [M+7H]^7+^ Calcd 628.4; Found 628.5 [M+6H]^6+^ Calcd 733.0; Found 733.0, [M+5H]^5+^ Calcd 879.4; Found 879.3, [M+4H]^4+^ Calcd 1099.0; Found 1098.9. The deconvoluted MS(ESI): Calcd for C_187_H_325_N_59_O_60_S_1_ 4392.0; Found 4391.9. Deconvoluted masses were obtained using the deconvolution function in Agilent ChemStation.

**Figure S18.** Synthesis of biotinylated N-terminal peptide of PAF15Ub2 (peptide **14**).　 **(A)** Synthetic scheme of peptide **14**. **(B)** One-pot NCL and deselenization. **8’** is a thioester of **8**. LC gradient: acetonitrile 1–40% over 20 min. In all HPLC column: ZORBAX 300SB-C8 3.5 µm (3.0 mm × 100 mm) (Agilent). Deconvoluted ESI–MS spectra were generated using UniDec.

**Figure S19**. ESI-MS spectrum of peptide **14**. Sequence: H−VRTKADSVPGTYRK(AGKQLEDGRTLSDYNIQKESTLHLVLRLRGG)VVAARAPRK(AGKQLEDGRTLSDYNIQKESTLHLVLRLRGG)VLGSSTSATNSTS−(spacer)−K(Biotin)−NH_2_, MS(ESI) *m/z*: [M+25H]^25+^ Calcd 860.4; Found 860.5, [M+24H]^24+^ Calcd 896.2; Found 896.4, [M+23H]^23+^ Calcd 935.1; Found 935.2, [M+22H]^22+^ Calcd 977.6; Found 977.6, [M+21H]^21+^ Calcd 1024.1; Found 1024.1, [M+20H]^20+^ Calcd 1075.3; Found 1075.3. The deconvoluted MS(ESI): Calcd for C_943_H_1579_N_269_O_294_S_3_ 21485.5; Found 21485.5. Deconvoluted masses were obtained using UniDec.

**Figure S20**. Preparation of DNMT1:PAF15Ub2:DNA^mCG/fCG^ ternary complex for cryo-EM. Size exclusion chromatography of the mixture of DNMT1, PAF15Ub2 peptide **8** and DNA^mCG/fCG^. Red box indicates the ternary complex used for cryo-EM analysis. Fractions corresponding to red box was analyzed by SDS-PAGE stained by Oriole^TM^ for detecting proteins (left) and Native-PAGE stained by Gelred^TM^ for detecting DNA (right).

**Figure S21**. Cryo-EM analysis of the DNMT1:PAF15Ub2:DNA^mCG/fCG^ ternary complex. **(A)** Cryo-EM processing and refinement workflow of the ternary complex. **(B)** FSC curve of the ternary complex was obtained (left). Local resolution of the ternary complex is shown (right).

**Figure S22**. Representative cryo-EM map of the DNMT1:PAF15Ub2:DNA^mCG/fCG^ ternary complex. **(A)** The atomic models of DNMT1 and DNA^mCG/fCG^ are superimposed on the cryo-EM map. **(B)** The structures around 5mC (left) and 5fC (right) in DNA^mCG/fCG^ are superimposed on the cryo-EM map.

**Figure S23**. **(A)** Cryo-EM map of Activating Helix and DNA Recognition Helix in the ternary complex superposed on cryo-EM map. DNA^mCG/fCG^ are shown as a transparent stick and cartoon model. **(B)** Structural comparison around the Toggle Pocket between DNMT1:PAF15Ub2:DNA^mCG/fCG^ and DNMT1:H3Ub2:DNA^mCG/fCG^ (PDB ID: 7XI9). Residues in Activating Helix that are involved in binding to the Toggle Pocket are shown as a stick models. DNMT1:PAF15Ub2:DNA^mCG/fCG^ and DNMT1:H3Ub2:DNA^mCG/fCG^ are colored in blue and orange, respectively.

3. **NMR spectra**

Boc-Sez-OH (**S1**)
